# Patritumab deruxtecan (HER3-DXd), a novel HER3 directed antibody drug conjugate, exhibits in vitro activity against breast cancer cells expressing HER3 mutations with and without HER2 overexpression

**DOI:** 10.1371/journal.pone.0267027

**Published:** 2022-05-03

**Authors:** Kumiko Koyama, Hirokazu Ishikawa, Manabu Abe, Yoshinobu Shiose, Suguru Ueno, Yang Qiu, Kenji Nakamaru, Masato Murakami

**Affiliations:** 1 Translational Science Department I, Daiichi Sankyo Co., Ltd., Tokyo, Japan; 2 Translational Research Department, Daiichi Sankyo RD Novare Co., Ltd., Tokyo, Japan; 3 Specialty Medicine Research Laboratories II, Daiichi Sankyo Co., Ltd., Tokyo, Japan; 4 Cell Therapy Research Laboratories, Daiichi Sankyo Co., Ltd., Tokyo, Japan; 5 Daiichi Sankyo, Inc., Basking Ridge, NJ, United States of America; Osmania University, Hyderabad, India, INDIA

## Abstract

ErbB3 (HER3), a member of the HER family, is overexpressed in various cancers and plays an important role in cell proliferation and survival. Certain HER3 mutations have also been identified as oncogenic drivers, making them potential therapeutic targets. In the current study, antitumor activity of patritumab deruxtecan (HER3-DXd), a HER3 directed antibody drug conjugate, was evaluated in tumor models with clinically reported HER3 mutations. MDA-MB-231, a HER3-negative human triple-negative breast cancer cell line, was transduced with lentiviral vectors encoding HER3 wild type (HER3^WT^), one of 11 HER3 mutations, or HER3 empty vector (HER3^EV^), in the presence/absence of HER2 overexpression. Targeted delivery of HER3-DXd was assessed using cell-surface binding, lysosomal trafficking, and cell-growth inhibition assays. HER3-DXd bound to the surface of HER3^WT^ and mutant cells in a similar, concentration-dependent manner but not to HER3^EV^. HER3-DXd was translocated to the lysosome, where time- and concentration-dependent signals were observed in the HER3 mutant and HER3^WT^ cells. HER3-DXd inhibited the growth of HER3^WT^ and HER3 mutant cells. HER3-DXd activity was observed in the presence and absence of HER2 overexpression. These data suggest that HER3-DXd may have activity against tumors expressing wild type HER3 or clinically observed HER3 mutations, supporting further clinical evaluation.

## Introduction

HER3 is a member of the human epidermal growth factor (EGFR/HER) family of receptor tyrosine kinases and is widely expressed in epithelial, neuronal, and mesenchymal cells [1]. HER3 is 1342 amino acids long with an extracellular domain (ECD) composed of four subdomains (I-IV), a single transmembrane domain, an intracellular kinase domain (KD), and a c-terminal regulatory region [2, 3]. HER3 is overexpressed in metastatic breast cancer, colorectal cancer, non–small cell lung cancer (NSCLC), and other tumor types [1, 2, 4]. HER3 has little intrinsic tyrosine kinase activity, but data suggest it frequently forms heterodimers with other receptor tyrosine kinases that can activate oncogenic signaling via the PI3K/Akt pathway and Src kinase, enhancing tumor cell survival, proliferation, and progression. HER3 can dimerize with other HER-family receptors, and it is a particularly potent partner for HER2, having a key role in its oncogenic activity [1, 4, 5]. In HER2 overexpressing breast cancers, HER3 results in more oncogenic activity with HER2 than with other HER receptors [1, 5]; HER2 and HER3 are commonly overexpressed together in breast cancers [4]. Findings from a meta-analysis showed that HER3 expression was associated with worse survival in patients with solid tumors, including colorectal, gastric, and breast [6]. Study findings have also shown that HER3 has a key role in resistance to cancer therapies [7]. Taken together, these findings identify HER3 as an attractive target for cancer therapy [1, 6].

Somatic mutations of EGFR and HER2 are well studied, and some are associated with the development and maintenance of the tumor as well as sensitivity to treatment [8–10]. The occurrence and functional relevance of HER3 mutations are of increasing interest to the scientific community given the importance of HER3 in HER2 signaling and the development of acquired therapeutic resistance [11]. Research shows that HER3 mutations are observed in many solid tumors, although they occur at a relatively low frequency; data suggest incidences of between 1% and 11% depending on the tumor type [3, 9, 11]. A search of cBioPortal in 2018 showed HER3 mutations in 1.05% of all breast cancers [3], and an incidence of 3.6% has been reported in patients with breast invasive lobular carcinoma [12]. Although a relatively high frequency of mutations has been reported in gastric (12%) and colorectal (11%) cancers, a lower frequency (1%) has been reported in NSCLC [11].

The majority of HER3 mutations identified are clustered in the ECD, with some mapping to the KD (S1 Fig) [9]. Most ECD mutations are at critical locations, such as the dimerization site or the border between two subdomains, possibly causing a shift toward the active confirmation of HER3 [11, 13]. Mutations in the KD of HER3 have been hypothesized to enhance interaction with its dimerization partners and/or elevate its phosphotransferase activity [3]. Certain HER3 mutants have demonstrated oncogenic driver activity in the absence or presence of HER2, and some can confer resistance to EGFR and HER2 directed therapies [9, 11, 13]. HER3 mutations are also reported to be a potential mechanism of tumor immune escape, as ectopic expression of mutated HER3 upregulated the expression of programmed-death ligand 1 by gallbladder carcinoma cells [14]. Overall, these data suggest a key role for HER3 mutations in the development, progression, and immune escape of tumors, making them an important consideration for HER3 directed treatment strategies [3].

Currently, there are no approved HER3 directed therapies for solid tumors, although various agents targeting HER3 using mono- and bispecific antibodies and small molecule inhibitors are under evaluation [2]. Antibody drug conjugates (ADCs) are an emerging class of cancer therapies that use a monoclonal antibody (mAb) to deliver a cytotoxic payload specifically to cells that express the mAb target antigen. The mechanism of action of ADCs is complex and may be payload-dependent or -independent; some ADCs can interfere with target function, dampen downstream signaling, and/or elicit antitumor immunity [15]. Patritumab deruxtecan (HER3-DXd, U3-1402) is a novel, investigational HER3 directed ADC that includes 3 key components: a fully human anti-HER3 immunoglobulin G1 (IgG1) mAb (patritumab), covalently linked to a topoisomerase I inhibitor payload (an exatecan derivative) via a tetrapeptide-based cleavable linker (S2 Fig). HER3-DXd has demonstrated preclinical antitumor efficacy in xenograft mouse models overexpressing HER3, with regression of tumors occurring without significant safety concerns [16]. HER3-DXd is being evaluated in clinical trials for the treatment of breast cancer and NSCLC, cancers that commonly express HER3 [17]. Preliminary data from a phase 1/2 study showed HER3-DXd had a manageable safety profile and clinically meaningful antitumor activity in heavily pretreated patients with hormone receptor-positive/HER2-negative metastatic breast cancer with high or low levels of HER3 expression (NCT02980341) [18]. Similarly, early data from a phase 1 trial in patients with metastatic/unresectable EGFR tyrosine kinase inhibitor (TKI)-resistant, *EGFR*-mutated NSCLC showed HER3-DXd had a manageable safety profile and clinically meaningful antitumor activity (NCT0326049) [19].

The objective of this preclinical study was to determine the functional relevance of clinically observed HER3 mutations with respect to HER3-DXd activity. The antitumor activity of HER3-DXd was evaluated in a breast cancer cell line transduced with 1 of 11 clinically reported HER3 mutations [9] in the presence or absence of HER2 overexpression.

## Materials and methods

### HER3 transduced cell lines

HER3 expressing cells were established from MDA-MB-231 (HER3-negative highly aggressive, invasive, and poorly differentiated human triple-negative breast cancer cell line [20]; ATCC Manassas, VA) at Daiichi Sankyo RD Novare Co, Ltd. MDA-MB-231 cells in the presence and absence of HER2 overexpression (established using a lentiviral vector [pLVSIN EF1α Neo] encoding full-length HER2) were infected with lentiviruses that were produced using vectors encoding FLAG-tagged full-length HER3 wild type (HER3^WT^), FLAG-tagged HER3 containing 1 of 11 HER3 mutations (V104L, V104M, A232V, P262H, G284R, D297Y, G325R, T355I, S846I, and E928G; S1 Fig) inserted into pLVSIN EF1α Pur (Takara cat. 6186), or vector without FLAG-tagged HER3 (HER3 empty vector or HER3^EV^). The HER3 Q809R mutant cell line was established only in the presence of HER2 overexpression.

### Experimental procedures

#### Cell-surface binding

The cell-surface binding of HER3-DXd (Daiichi Sankyo Co., Ltd.) to the HER3 transfectants was assessed by flow cytometry using a phycoerythretin anti-FLAG antibody (BioLegend, San Diego, CA). Cells were harvested as cell suspension, and then incubated on ice for 1 hour with 0.1, 1, 10, and 100 nM HER3-DXd in stain buffer. The cells were washed twice with stain buffer and resuspended in secondary antibody, 10 μg/mL Alexa Fluor 647 goat antihuman IgG in stain buffer, and incubated on ice for 1 hour under dark conditions. The cells were washed twice with stain buffer and then analyzed by flow cytometer, Attune NxT (Thermo Fisher Scientific Inc., Waltham, MA). Cell-surface binding was determined using FlowJo™ software (version 10; BD Life Sciences, Ashland, OR) and expressed as the mean fluorescence intensity (MFI) of the cells that were stained positive for HER3-DXd. The MFI of an unstained sample was used as background and subtracted from the result for each stained sample. A FLAG-negative cell line was used as a negative control. Cell-surface HER2 expression level was assessed in HER3^EV^ cells with and without the transduction of the lentiviral vector encoding HER2 by flow cytometry, by incubating with 100 nM trastuzumab deruxtecan (DS-8201a; Daiichi Sankyo Co., Ltd.) as an anti-HER2 antibody.

#### Lysosomal trafficking

The lysosomal trafficking of 0.1, 1, and 10 nM HER3-DXd into the HER3 transfectants was determined using HER3-DXd labeled with a pH-sensitive dye, pHrodo (pHrodo iFL Red Microscale Protein Labeling Kit #P36014, Thermo Fisher Scientific Inc). The cells were treated with the pHrodo-labelled HER3-DXd, 100 nM LysoTracker Green DND-26, and 100 ng/mL Hoechst 33342 (both Thermo Fisher Scientific Inc). Fluorescence emission images were then collected at 30-minute intervals for up to 12 hours using a 403 confocal Opera Phenix high-content screening system (PerkinElmer Co., Ltd., Waltham, MA). Using Harmony analysis software (PerkinElmer Co., Ltd.), the level of lysosomal trafficking was expressed as the number of dots per cell and the dot signal intensity. Dot signal intensity was calculated by subtracting the average dot intensity value at t = 0 from each signal intensity value. The level of lysosomal trafficking was also expressed as a trafficking index, calculated by multiplying the number of dots per cell by the delta dot signal intensity.

#### Cell-growth inhibition

The effects of 10 nM of HER3-DXd, patritumab, IgG-ADC (Daiichi Sankyo Co., Ltd.) [16], or DXd (MAAA-1181d) payload (Daiichi Sankyo Co., Ltd.) on the growth of the HER3 transfectants after 6 days of incubation were assessed in triplicate or more by measuring the amount of ATP using CellTiter-Glo^®^ (Promega Corporation, Madison, WI) luminescent cell viability assay, using EnVision multimode plate reader (PerkinElmer Inc.). Cell viability was calculated as the luminescence intensity of the test well divided by the MFI of untreated wells multiplied by 100. A *t*-test was performed using SAS System Release 9.2 (SAS Institute, Inc, Cary, NC) to compare whether the treatment groups (HER3-DXd, patritumab, and DXd) were different from the control group.

## Results and discussion

### Cell-surface binding

**Fig 1:**
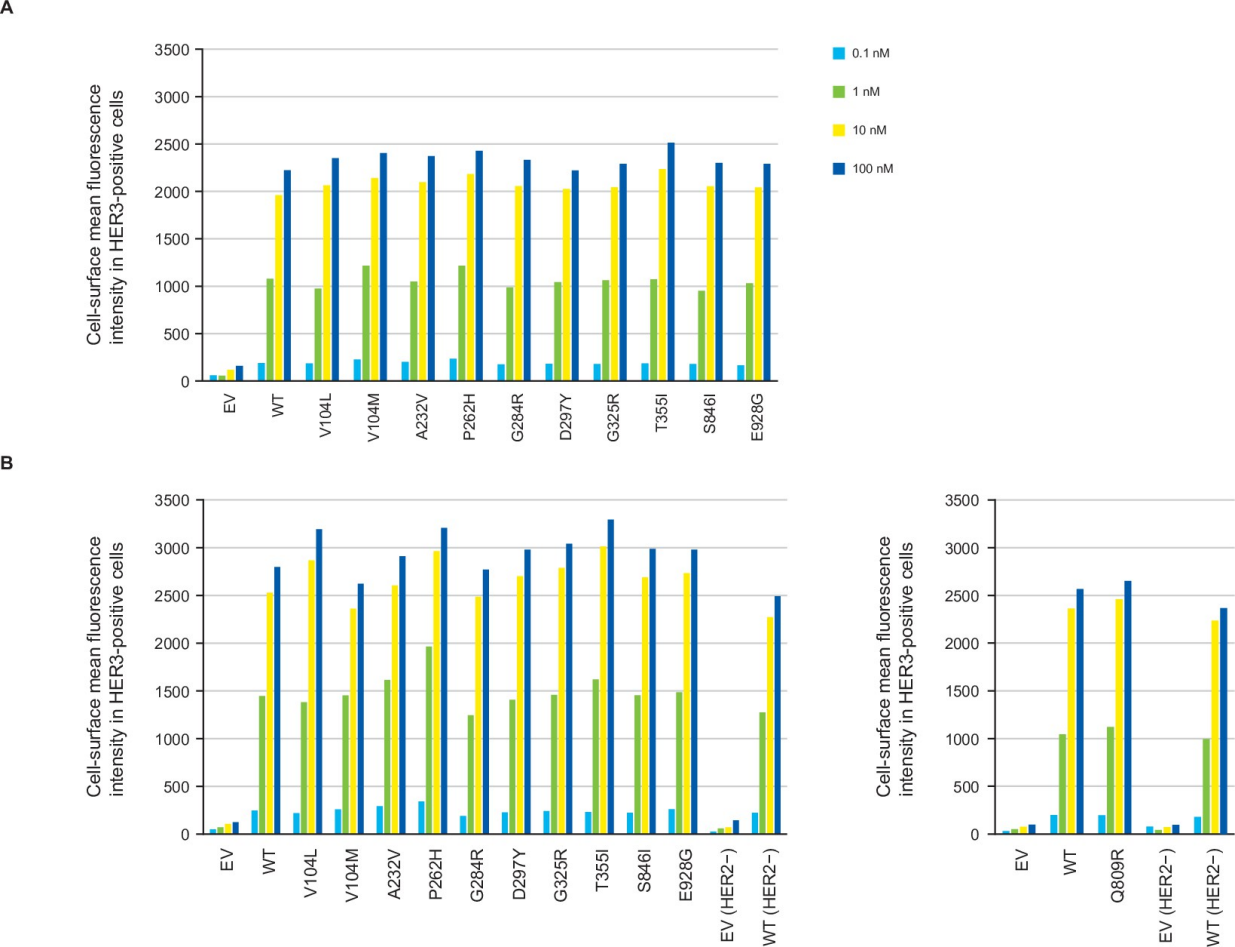
Cell-surface binding of HER3-DXd in MDA-MB-231 cells transduced with HER3^WT^, HER3 mutations, or HER3^EV^ in the absence (A) or presence (B) of HER2 overexpression. Cells were incubated with 0.1, 1, 10, and 100 nM HER3-DXd, followed by secondary antibody (10 mg/mL Alexa Fluor 647 goat antihuman IgG). The cell-surface binding of HER3-DXd was assessed by flow cytometry and expressed as the mean fluorescence intensity of the cells that were stained positive for HER3-DXd. Abbreviations: EV = empty vector, HER = human epidermal growth factor receptor, WT = wild type.

HER3-DXd bound to the surface of all HER3-mutant and HER3^WT^ cells in a concentration-dependent manner (Fig 1). The amount of HER3-DXd bound to the surface of each of the HER3 mutant cells was similar to the amount bound to the HER3^WT^ cells at all the concentrations tested, with binding saturation being reached at around 10 nM. HER3-DXd did not bind to the surface of HER3^EV^ cells. The overexpression of HER2 had little or no impact on the cell-surface binding of HER3-DXd to the various transduced cells (Fig 1), under the levels of HER2 overexpression as shown in S3 Fig. A phycoerythretin anti-FLAG antibody was used to qualitatively check FLAG-tagged HER3 expression levels by lentiviral transduction. The MFI values in the transfectants without HER2 overexpression (HER2–) ranged from 305 to 405, whereas MFI for transfectants with HER2 overexpression (HER2+) ranged from 371 to 492. Both assays were conducted on different days, using HER3^WT^ HER2– cells as a control. The MFI of this control was slightly different between the assays: 305 for the former (HER2–) and 376 for the latter (HER2+). Therefore, we concluded that the difference in transfectant efficiency was negligible, and no normalization was conducted.

### Lysosomal trafficking

**Fig 2:**
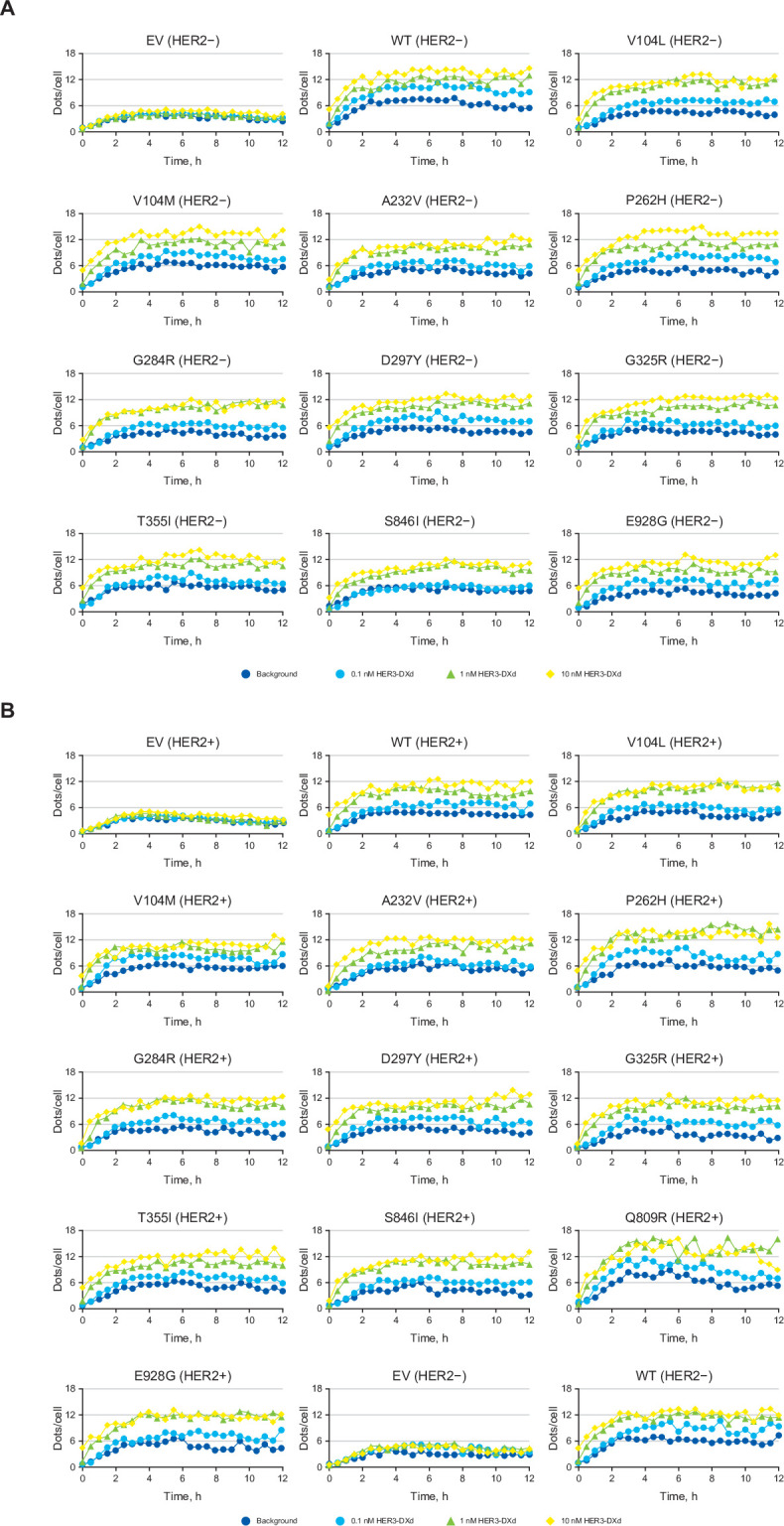
Trafficking (dots per cell) of pHrodo-labeled HER3-DXd in MDA-MB-231 cells transduced with HER3^WT^, HER3 mutations, or HER3^EV^ in the absence (A) or presence (B) of HER2 overexpression. The lysosomal trafficking of 0.1, 1, and 10 nM HER3-DXd into the HER3 transfectants was determined using HER3-DXd labeled with pHrodo. Fluorescence emission images were then collected at 30-minute intervals for up to 12 hours. The level of lysosomal trafficking was expressed as the number of dots per cell. Abbreviations: EV = empty vector, HER = human epidermal growth factor receptor, WT = wild type.

HER3-DXd was translocated to the lysosome where pHrodo-labeled HER3-DXd–derived signals were observed in a time- and concentration-dependent manner at comparable levels in the HER3^WT^ and all HER3-mutant cells. HER3-DXd trafficking was higher in the HER3^WT^ than in the HER3^EV^ cells (representative trafficking [dots per cell] data are shown Fig 2; see S4 Fig for additional trafficking results). Representative time-lapse images for the trafficking of pHrodo-labeled HER3-DXd in HER3^WT^, HER3-mutant, and HER3^EV^ cells are shown in S5 Fig. HER2 overexpression had little or no significant impact on the trafficking of HER3-DXd.

### Cell-growth inhibition

**Fig 3:**
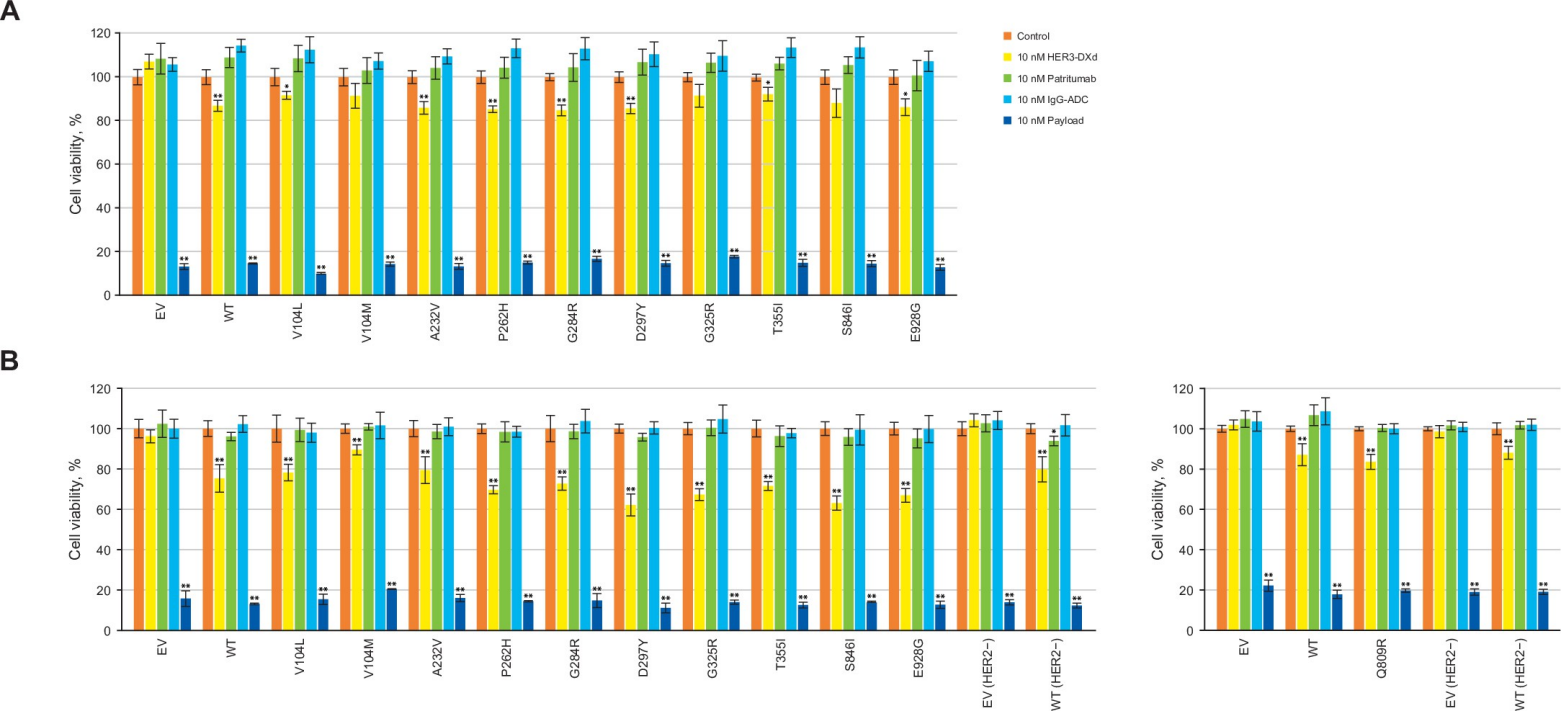
Cell-growth inhibition activity of HER3-DXd, patritumab, and payload against MDA-MB-231 cells transduced with HER3^WT^, HER3 mutations, and HER3^EV^ in the absence (A) or presence (B) of HER2 overexpression.^a^ Mean ± SD. *p<0.01, **p<0.001. The effects of 10 nM of HER3-DXd, patritumab, or DXd payload on the growth of HER3 transfectants after 6 days of incubation were assessed and statistically compared with the control. Cell viability was calculated as the luminescence intensity of the test well divided by the mean luminescence intensity of untreated wells multiplied by 100. IgG-ADC is the negative control. Abbreviations: ADC = antibody drug conjugate, EV = empty vector, HER = human epidermal growth factor receptor, IgG = immunoglobulin G, SD = standard deviation, WT = wild type. ^a^All data in part A are mean quadruplicates. In part B, all data are quintuplicates except for 10 nM payload, which is triplicate.

Based on cell-surface binding and lysosomal trafficking data (Figs 1 and 2), 10 nM HER3-DXd, IgG-ADC (negative control), patritumab, or DXd were used to assess growth-inhibitory activity in HER3^WT^, HER3-mutant, and HER3^EV^ cells (Fig 3). HER3-DXd showed growth-inhibitory activity against HER3^WT^ (mean cell viability: 86.9%, standard deviation [SD]: 2.5; *p*<0.001) and most HER3-mutant cells (mean cell viability [SD] ranged from 84.8% [2.5%] to 92.2% [3.2%]; *p* values <0.01 or <0.001). HER3-DXd had no inhibitory activity against HER3^EV^ cells. The impact on growth inhibition (via mean cell viability) was similar in the presence or absence of HER2 overexpression but this impact was not found to be substantial in cells expressing HER3 mutants V104M, G325R, and S846I in the absence of HER2 overexpression. Payload alone had significant growth-inhibitory activity against the HER3^WT^ and HER3-mutant cells (*p*<0.001); patritumab had no inhibitory activity.

The findings from this study showed that HER3-DXd achieved effective payload delivery via cell-surface binding and lysosomal trafficking, resulting in cell-growth inhibition. HER3-DXd had activity irrespective of HER3 mutation status, with substantial inhibition observed against tumor cells expressing HER3^WT^ and many of the clinically identified HER3 mutations. The cell-surface binding data demonstrate that HER3-DXd has a HER3-specific cell-surface binding property, and the binding affinity of HER3-DXd appears comparable regardless of whether mutated or wild-type HER3 is expressed. After cell binding, HER3-DXd is translocated to the lysosome, with data showing that this occurs in a time- and concentration-dependent manner in all the HER3-mutant and HER3WT cells tested but not in HER3^EV^ cells. Payload alone also inhibited the growth of HER3WT cells, whereas patritumab alone and IgG-ADC alone had no activity. These data suggest that the cell growth-inhibition activity of HER3-DXd is dependent on the payload released within the cells, after the binding to cell-surface HER3 and the trafficking to lysosome.

HER3 mutations are emerging as an important consideration for determining the optimal approach to treating many cancers. In vitro data from HER3-transformed colon and breast tumor cell lines showed that HER3 mutants in the ECD (V104M, A232V, P262H, G284R, T389K) and KD (Q809R, S846I, E928G) promoted tumor cell growth. This activity was ligand independent but required HER2 expression [11]. These data suggest HER2 expression is necessary for the oncogenic activity of HER3 mutation, as may be expected given the impaired kinase activity of HER3. However, more recent data showed that in estrogen receptor-positive breast cancer cells, the T355I ECD mutation was activating and increased cellular proliferation in the absence of HER2 [13]. It is hypothesized that the T355I mutation shifts HER3 to an active conformation, promoting dimerization with the HER family receptors EGFR and HER4. Given this, agents with activity against HER3 mutations may be beneficial because targeting HER2 may not affect the oncogenic activity associated with some HER3 mutations. Our data demonstrated the ability of HER3-DXd to bind to the cell surface, traffic through the cell, and inhibit cell growth with or without the presence of HER2 overexpression.

The role of HER3 mutations in treatment resistance is of particular clinical interest and requires further study. In vitro data generated using a breast cancer cell line showed that lapatinib, a dual EGFR/HER2 TKI, reduced the proliferation of cells expressing HER3^WT^ or HER^EV^, but it did not affect cells expressing HER3 with mutations in the ECD (F94L, G284R, D297Y, T355I) or KD (E1261A) [13]. In the SUMMIT study of the pan-HER TKI neratinib for patients with various types of solid tumors, neratinib had no antitumor activity in any of the 16 patients expressing mutated HER3 [21]. Although limited, these data suggest that the currently approved HER TKIs may not be effective against all HER3 mutations, and novel treatment strategies may be required for some patients.

## Conclusion

In summary, findings from this study demonstrate that efficient payload delivery via ADC-mediated internalization can be achieved across different clinically observed HER3 mutations. The investigational HER3 directed ADC, HER3-DXd, exhibited activity in vitro against cells overexpressing HER3 and many of the assessed HER3 mutations, and this activity was observed in the presence and absence of HER2 overexpression. These data suggest that HER3-DXd could exhibit antitumor activity in patients irrespective of the presence or absence of clinically relevant HER3 mutations, supporting continued evaluation. Seven clinical trials are in progress to evaluate HER3-DXd for the treatment of patients with breast cancer or NSCLC [17]; these are tumors that are known to overexpress HER3 and in which HER3 mutations have been reported [1, 3, 4].

## Supporting information

S1 Fig Distribution of HER3 somatic mutations. (Adapted from Mishra R, Hankler AB, Garrett JT. Genomic alterations of ERBB receptors in cancer: clinical implications. *Oncotarget*. 2017;8:114371–92. This work is licensed under a Creative Commons Attribution 3.0 International (CC BY 3.0) License. https://creativecommons.org/licenses/by/3.0/) [1]. Abbreviation: aa = amino acids.
(DOCX)

S2 Fig Structure of HER3-DXd [1–4]. (DOCX)

S3 Fig Cell-surface HER2 expression in MDA-MB-231 cells with (EV [HER2+]) and without (EV [HER2–]) HER2 overexpression. Cells were incubated with 100 nM trastuzumab deruxtecan, an antibody drug conjugate comprised of an anti-HER2 antibody, followed by secondary antibody (10 μg/mL Alexa Fluor 647 goat antihuman IgG). The cell-surface HER2 expression level was assessed by flow cytometry. Abbreviations: EV = empty vector, HER = human epidermal growth factor receptor.
(DOCX)

S4 Fig Trafficking (trafficking index) of pHrodo-labeled HER3-DXd in MDA-MB-231 cells transduced with HER3WT, HER3 mutations, or HER3EV in the absence (A) or presence (B) of HER2 overexpression. The lysosomal trafficking of 0.1, 1, and 10 nM HER3-DXd into the HER3 transfectants was determined using HER3-DXd labeled with pHrodo. Fluorescence emission images were then collected at 30-minute intervals for up to 12 hours. The level of lysosomal trafficking was expressed as a trafficking index (number of dots per cell multiplied by the delta dot signal intensity). Abbreviations: EV = empty vector, HER = human epidermal growth factor receptor, WT, wild type.
(DOCX)

S5 Fig Representative images for trafficking of pHrodo-labeled HER3-DXd in MDA-MB-231 cells transduced with HER3WT, HER3 mutations, and HER3EV in the absence (A) or presence (B) of HER2 overexpression. Cell nuclei were labeled with Hoechst (blue), and images of live cells were taken every 30 minutes after the addition of pHrodo-labeled HER3-DXd (red). Images represent cells 6 hours after addition of 1 nM pHrodo-labeled HER3-DXd and were taken with a ×63 water immersion objective lens. Abbreviations: EV = empty vector, HER = human epidermal growth factor receptor, WT = wild type.
(DOCX)

S1 Table Cell surface mean fluorescence intensity (MFI) values for HER3-DXd in MDA-MB-231 cells transduced with lentiviral vectors encoding flag-tagged HER3 wild-type or HER3 mutations. (PDF)

S2 Table Trafficking (dots/cell) of pHrodo-labeled HER3-DXd in MDA-MB-231 cells transduced with lentiviral vectors encoding flag-tagged HER3 wild-type or HER3 mutations. (PDF)

S3 Table Trafficking index (dots/cell x delta dot signal intensity) of pHrodo-labeled HER3-DXd in MD-MB-231 cells transduced with lentiviral vectors encoding flag-tagged HER3 wild-type or HER3 mutations. (PDF)

S4 Table Individual cell growth inhibition activity of HER3-DXd, patritumab, IgG-ADC, and payload against MDA-MB-231 cells transduced with lentiviral vectors encoding flag-tagged HER3 wild-type or HER3 mutations. (PDF)
