# Supplementary material for: Patritumab deruxtecan (HER3-DXd), a novel HER3 directed antibody drug conjugate, exhibits in vitro activity against breast cancer cells expressing HER3 mutations with and without HER2 overexpression
Source: PLoS One. 2022 May 3;17(5):e0267027. doi: 10.1371/journal.pone.0267027 (PMC9064083; doi:10.1371/journal.pone.0267027)
Supplement: S1 Fig — (Adapted from Mishra R, Hankler AB, Garrett JT. Genomic alterations of ERBB receptors in cancer: clinical implications. Oncotarget. 2017;8:114371–92. This work is licensed under a Creative Commons Attribution 3.0 International (CC BY 3.0) License. https://creativecommons.org/licenses/by/3.0/) [1]. Abbreviation: aa = amino acids. (DOCX) [file pone.0267027.s001.docx]

**S1 Fig. Distribution of HER3 somatic mutations** (Adapted from Mishra R, Hankler AB, Garrett JT. Genomic alterations of ERBB receptors in cancer: clinical implications. *Oncotarget.* 2017;8:114371–92. This work is licensed under a Creative Commons Attribution 3.0 International (CC BY 3.0) License. <https://creativecommons.org/licenses/by/3.0/>) [1]. Abbreviation: aa = amino acids.


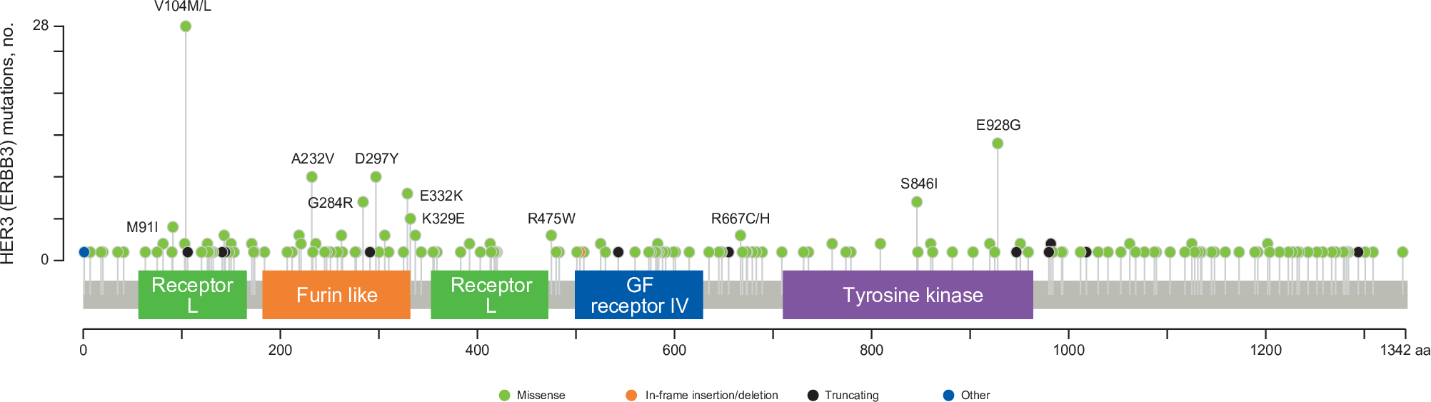


**Reference**

1. Mishra R, Hanker AB, Garrett JT. Genomic alterations of ERBB receptors in cancer: clinical implications. Oncotarget. 2017;8: 114371-114392.
