# Supplementary material for: Patritumab deruxtecan (HER3-DXd), a novel HER3 directed antibody drug conjugate, exhibits in vitro activity against breast cancer cells expressing HER3 mutations with and without HER2 overexpression
Source: PLoS One. 2022 May 3;17(5):e0267027. doi: 10.1371/journal.pone.0267027 (PMC9064083; doi:10.1371/journal.pone.0267027)
Supplement: S2 Fig — (DOCX) [file pone.0267027.s002.docx]

**S2 Fig. Structure of HER3-DXd** [1-4].


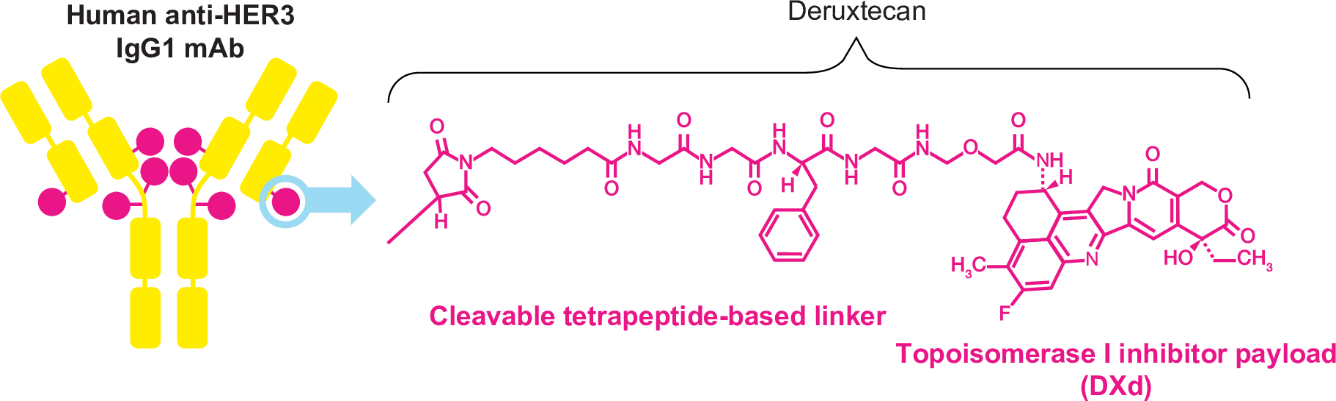


**References**

1. Hashimoto Y, Koyama K, Kamai Y, Hirotani K, Ogitani Y, Zembutsu A, et al. A novel HER3-targeting antibody-drug conjugate, U3-1402, exhibits potent therapeutic efficacy through the delivery of cytotoxic payload by efficient internalization. Clin Cancer Res. 2019;25: 7151-7161.

2. Nakada T, Sugihara K, Jikoh T, Abe Y, Agatsuma T. The latest research and development into the antibody-drug conjugate, [fam-] trastuzumab deruxtecan (DS-8201a), for HER2 cancer therapy. Chem Pharm Bull (Tokyo). 2019;67: 173-185.

3. Ogitani Y, Aida T, Hagihara K, Yamaguchi J, Ishii C, Harada N, et al*.* DS-8201a, a novel HER2-targeting ADC with a novel DNA topoisomerase I inhibitor, demonstrates a promising antitumor efficacy with differentiation from T-DM1. Clin Cancer Res. 2016;22: 5097-5108.

4. Koganemaru S, Kuboki Y, Koga Y, Kojima T, Yamauchi M, Maeda N, et al*.* U3-1402, a novel HER3-targeting antibody-drug conjugate, for the treatment of colorectal cancer. Mol Cancer Ther. 2019;18: 2043-2050.
