# Supplementary material for: Patritumab deruxtecan (HER3-DXd), a novel HER3 directed antibody drug conjugate, exhibits in vitro activity against breast cancer cells expressing HER3 mutations with and without HER2 overexpression
Source: PLoS One. 2022 May 3;17(5):e0267027. doi: 10.1371/journal.pone.0267027 (PMC9064083; doi:10.1371/journal.pone.0267027)

**S3 Fig. Cell-surface HER2 expression in MDA-MB-231 cells with (EV [HER2+]) and without (EV [HER2–]) HER2 overexpression.** Cells were incubated with 100 nM trastuzumab deruxtecan, an antibody drug conjugate comprised of an anti-HER2 antibody, followed by secondary antibody (10 μg/mL Alexa Fluor 647 goat antihuman IgG). The cell-surface HER2 expression level was assessed by flow cytometry. Abbreviations: EV = empty vector, HER = human epidermal growth factor receptor.


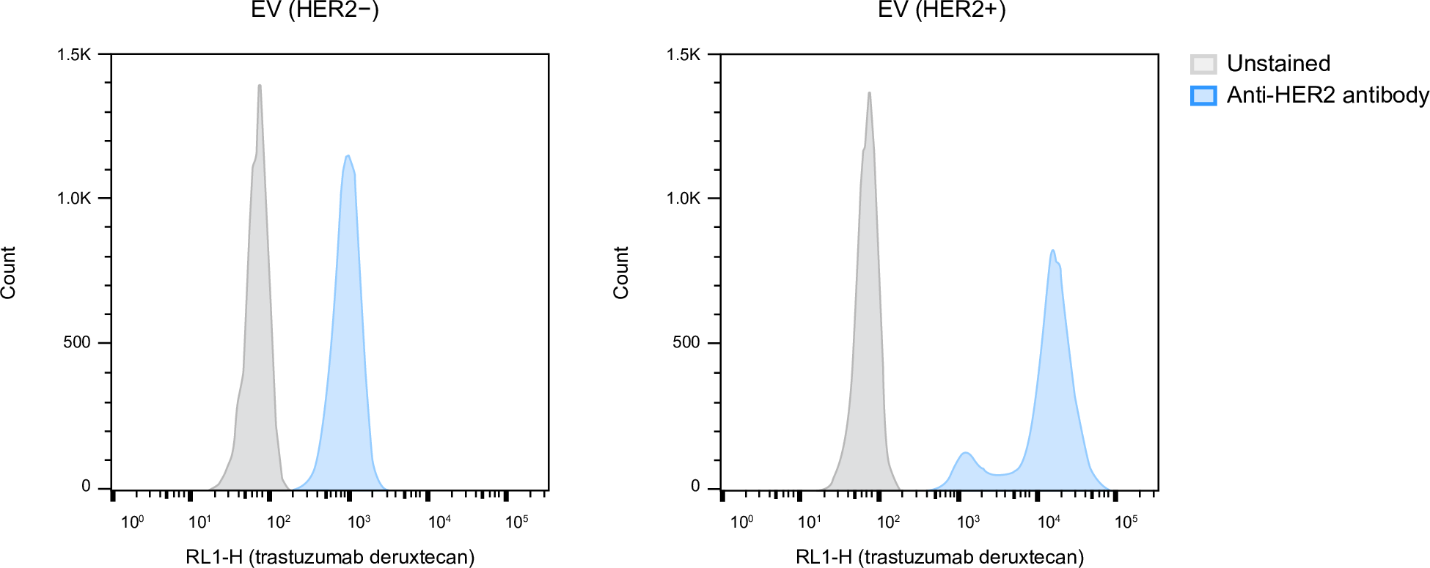

Supplement: S3 Fig — Cells were incubated with 100 nM trastuzumab deruxtecan, an antibody drug conjugate comprised of an anti-HER2 antibody, followed by secondary antibody (10 μg/mL Alexa Fluor 647 goat antihuman IgG). The cell-surface HER2 expression level was assessed by flow cytometry. Abbreviations: EV = empty vector, HER = human epidermal growth factor receptor. (DOCX) [file pone.0267027.s003.docx]
