# Supplementary material for: Patritumab deruxtecan (HER3-DXd), a novel HER3 directed antibody drug conjugate, exhibits in vitro activity against breast cancer cells expressing HER3 mutations with and without HER2 overexpression
Source: PLoS One. 2022 May 3;17(5):e0267027. doi: 10.1371/journal.pone.0267027 (PMC9064083; doi:10.1371/journal.pone.0267027)

**S4 Fig. Trafficking (trafficking index) of pHrodo-labeled HER3-DXd in MDA-MB-231 cells transduced with HER3^WT^, HER3 mutations, or HER3^EV^ in the absence (A) or presence (B) of HER2 overexpression.** The lysosomal trafficking of 0.1, 1, and 10 nM HER3-DXd into the HER3 transfectants was determined using HER3-DXd labeled with pHrodo. Fluorescence emission images were then collected at 30-minute intervals for up to 12 hours. The level of lysosomal trafficking was expressed as a trafficking index (number of dots per cell multiplied by the delta dot signal intensity). Abbreviations: EV = empty vector, HER = human epidermal growth factor receptor, WT, wild type


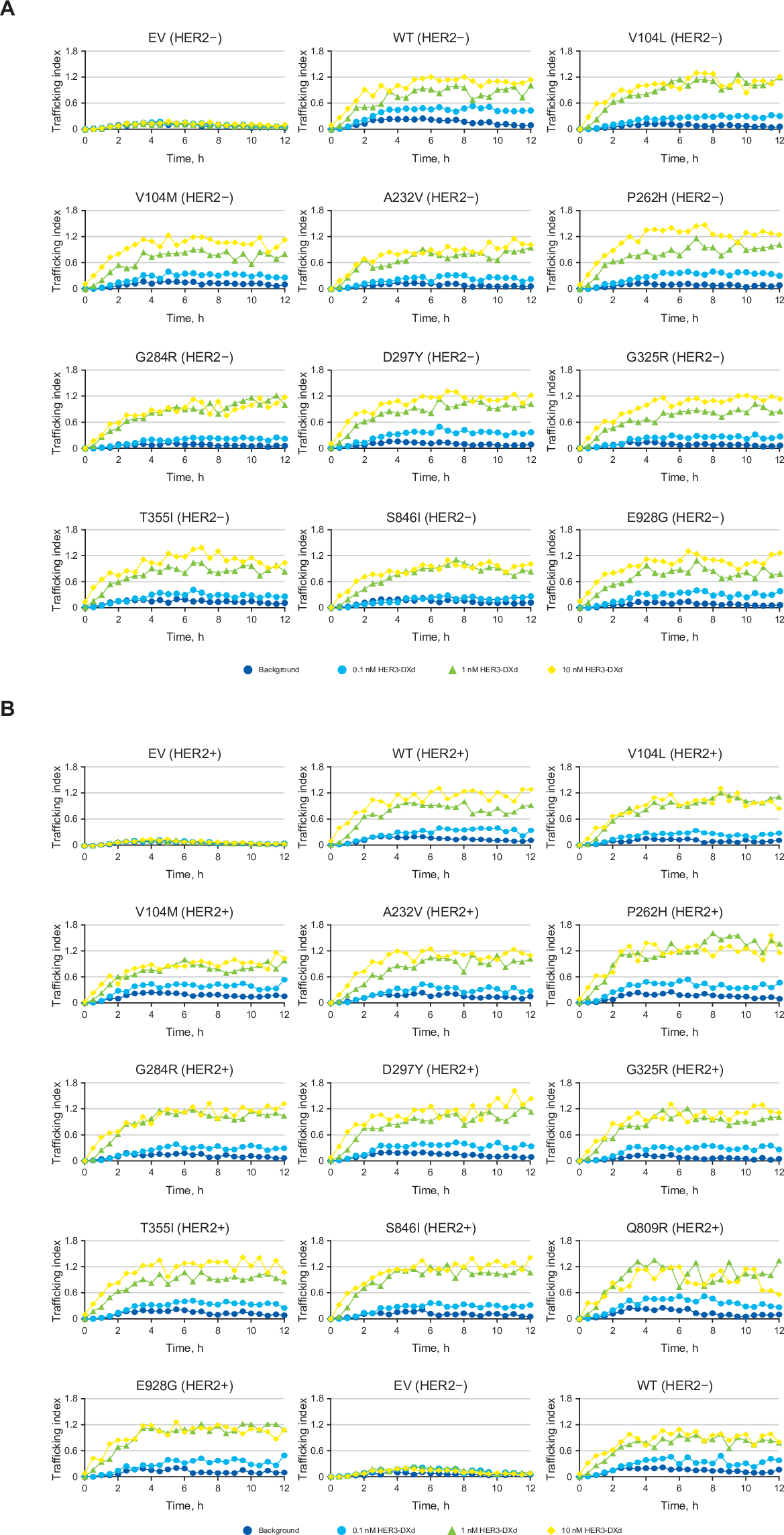

Supplement: S4 Fig — Trafficking (trafficking index) of pHrodo-labeled HER3-DXd in MDA-MB-231 cells transduced with HER3WT, HER3 mutations, or HER3EV in the absence (A) or presence (B) of HER2 overexpression. The lysosomal trafficking of 0.1, 1, and 10 nM HER3-DXd into the HER3 transfectants was determined using HER3-DXd labeled with pHrodo. Fluorescence emission images were then collected at 30-minute intervals for up to 12 hours. The level of lysosomal trafficking was expressed as a trafficking index (number of dots per cell multiplied by the delta dot signal intensity). Abbreviations: EV = empty vector, HER = human epidermal growth factor receptor, WT, wild type. (DOCX) [file pone.0267027.s004.docx]
