# Supplementary material for: Patritumab deruxtecan (HER3-DXd), a novel HER3 directed antibody drug conjugate, exhibits in vitro activity against breast cancer cells expressing HER3 mutations with and without HER2 overexpression
Source: PLoS One. 2022 May 3;17(5):e0267027. doi: 10.1371/journal.pone.0267027 (PMC9064083; doi:10.1371/journal.pone.0267027)

**S5 Fig. Representative images for trafficking of pHrodo-labeled HER3-DXd in MDA-MB-231 cells transduced with HER3^WT^, HER3 mutations, and HER3^EV^ in the absence (A) or presence (B) of HER2 overexpression.** Cell nuclei were labeled with Hoechst (blue), and images of live cells were taken every 30 minutes after the addition of pHrodo-labeled HER3-DXd (red). Images represent cells 6 hours after addition of 1 nM pHrodo-labeled HER3-DXd and were taken with a ×63 water immersion objective lens. Abbreviations: EV = empty vector, HER = human epidermal growth factor receptor, WT = wild type.


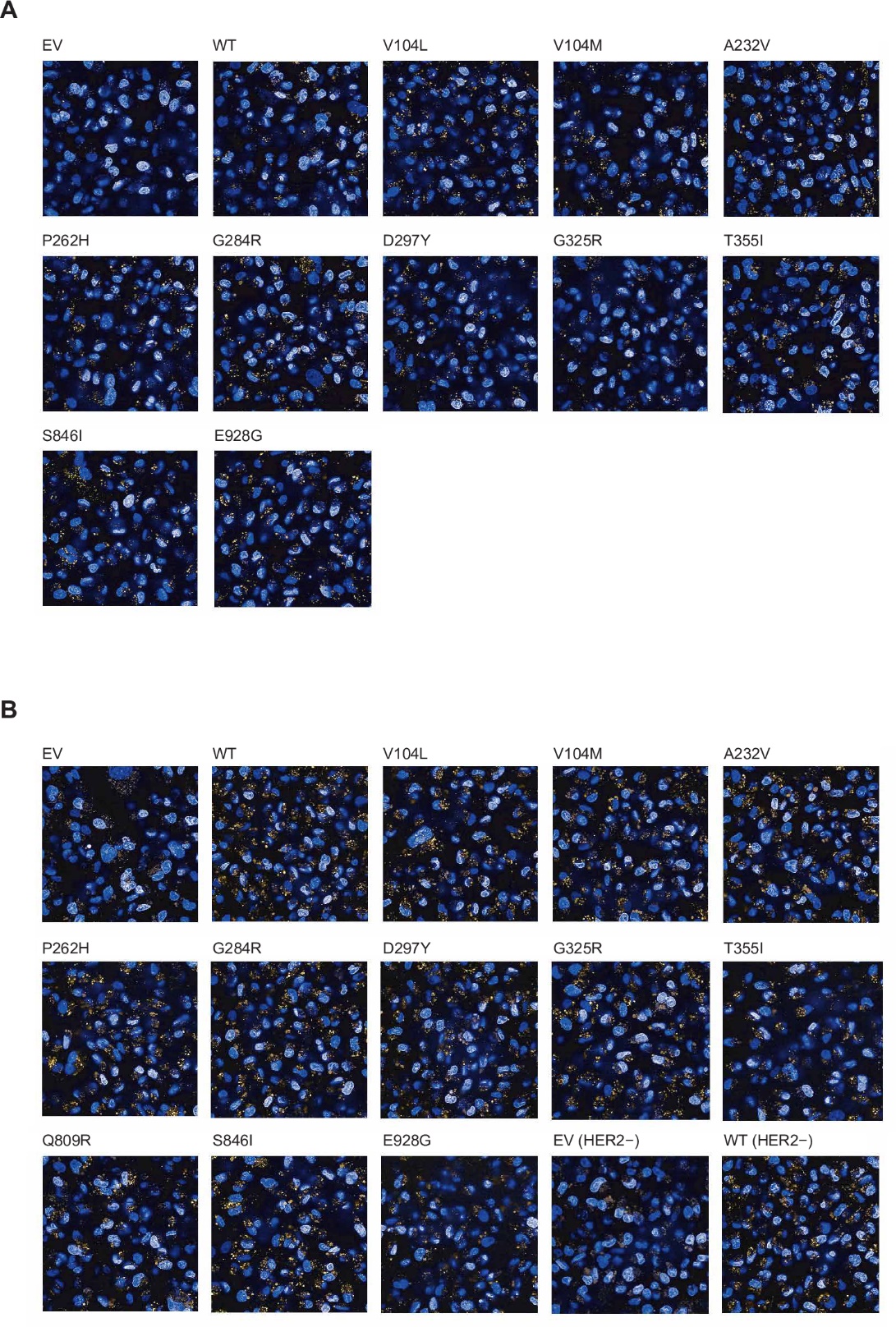

Supplement: S5 Fig — Representative images for trafficking of pHrodo-labeled HER3-DXd in MDA-MB-231 cells transduced with HER3WT, HER3 mutations, and HER3EV in the absence (A) or presence (B) of HER2 overexpression. Cell nuclei were labeled with Hoechst (blue), and images of live cells were taken every 30 minutes after the addition of pHrodo-labeled HER3-DXd (red). Images represent cells 6 hours after addition of 1 nM pHrodo-labeled HER3-DXd and were taken with a ×63 water immersion objective lens. Abbreviations: EV = empty vector, HER = human epidermal growth factor receptor, WT = wild type. (DOCX) [file pone.0267027.s005.docx]
