# Supplementary material for: Patritumab deruxtecan (HER3-DXd), a novel HER3 directed antibody drug conjugate, exhibits in vitro activity against breast cancer cells expressing HER3 mutations with and without HER2 overexpression
Source: PLoS One. 2022 May 3;17(5):e0267027. doi: 10.1371/journal.pone.0267027 (PMC9064083; doi:10.1371/journal.pone.0267027)
Supplement: S1 Table — (PDF) [file pone.0267027.s006.pdf]

A) HER2 overexpression (-)

HER3	Cell surface MFI in HER3 positive cells					Corrected cell surface MFI in HER3 positive cells				
	Unstained	0.1 nM HER3-DXd	1 nM HER3-DXd	10 nM HER3-DXd	100 nM HER3-DXd	Unstained	0.1 nM HER3-DXd	1 nM HER3-DXd	10 nM HER3-DXd	100 nM HER3-DXd
EV	70.3	130	127	188	231	0	59.7	56.7	117.7	160.7
WT	66.6	256	1145	2027	2290	0	189.4	1078.4	1960.4	2223.4
V104L	67.3	252	1043	2130	2418	0	184.7	975.7	2062.7	2350.7
V104M	66.7	294	1283	2209	2472	0	227.3	1216.3	2142.3	2405.3
A232V	66.6	267	1115	2165	2439	0	200.4	1048.4	2098.4	2372.4
P262H	66.6	301	1284	2251	2494	0	234.4	1217.4	2184.4	2427.4
G284R	67.1	242	1055	2123	2399	0	174.9	987.9	2055.9	2331.9
D297Y	66.7	249	1109	2092	2289	0	182.3	1042.3	2025.3	2222.3
G325R	66.7	246	1131	2111	2356	0	179.3	1064.3	2044.3	2289.3
T355I	66.5	253	1140	2301	2580	0	186.5	1073.5	2234.5	2513.5
S846I	69.9	249	1021	2124	2371	0	179.1	951.1	2054.1	2301.1
E928G	66.5	232	1098	2109	2357	0	165.5	1031.5	2042.5	2290.5

Corrected cell surface MFI was subtracted by unstained value.

B) HER2 overexpression (+)

HER3	Cell surface MFI in HER3 positive cells					Corrected cell surface MFI in HER3 positive cells				
	Unstained	0.1 nM HER3-DXd	1 nM HER3-DXd	10 nM HER3-DXd	100 nM HER3-DXd	Unstained	0.1 nM HER3-DXd	1 nM HER3-DXd	10 nM HER3-DXd	100 nM HER3-DXd
EV	68.6	118	139	176	194	0	49.4	70.4	107.4	125.4
WT	67.9	314	1514	2597	2864	0	246.1	1446.1	2529.1	2796.1
V104L	69.3	288	1451	2936	3263	0	218.7	1381.7	2866.7	3193.7
V104M	69.8	330	1522	2431	2691	0	260.2	1452.2	2361.2	2621.2
A232V	69.4	361	1683	2674	2979	0	291.6	1613.6	2604.6	2909.6
P262H	69.3	412	2032	3033	3276	0	342.7	1962.7	2963.7	3206.7
G284R	69	259	1313	2554	2840	0	190.0	1244.0	2485.0	2771.0
D297Y	69.1	298	1476	2770	3048	0	228.9	1406.9	2700.9	2978.9
G325R	68.9	310	1526	2857	3110	0	241.1	1457.1	2788.1	3041.1
T355I	69.2	302	1689	3083	3363	0	232.8	1619.8	3013.8	3293.8
S846I	69.3	293	1524	2758	3057	0	223.7	1454.7	2688.7	2987.7
E928G	69.6	331	1556	2803	3048	0	261.4	1486.4	2733.4	2978.4
EV (HER2-)	70.5	97.8	130	143	214	0	27.3	59.5	72.5	143.5
WT (HER2-)	68.2	291	1344	2341	2560	0	222.8	1275.8	2272.8	2491.8

Corrected cell surface MFI was subtracted by unstained value.

C) HER2 overexpression (+), Q809R

HER3	Cell surface MFI in HER3 positive cells					Corrected cell surface MFI in HER3 positive cells				
	Unstained	0.1 nM HER3-DXd	1 nM HER3-DXd	10 nM HER3-DXd	100 nM HER3-DXd	Unstained	0.1 nM HER3-DXd	1 nM HER3-DXd	10 nM HER3-DXd	100 nM HER3-DXd
EV	64.3	96	115	141	162	0	31.7	50.7	76.7	97.7
WT	63.3	263	1107	2427	2631	0	199.7	1043.7	2363.7	2567.7
Q809R	63.5	261	1185	2523	2716	0	197.5	1121.5	2459.5	2652.5
EV (HER2-)	65.1	143	108	137	161	0	77.9	42.9	71.9	95.9
WT (HER2-)	62.4	241	1057	2298	2428	0	178.6	994.6	2235.6	2365.6

Corrected cell surface MFI was subtracted by unstained value.
