# Supplementary material for: Patritumab deruxtecan (HER3-DXd), a novel HER3 directed antibody drug conjugate, exhibits in vitro activity against breast cancer cells expressing HER3 mutations with and without HER2 overexpression
Source: PLoS One. 2022 May 3;17(5):e0267027. doi: 10.1371/journal.pone.0267027 (PMC9064083; doi:10.1371/journal.pone.0267027)
Supplement: S2 Table — (PDF) [file pone.0267027.s007.pdf]

A) HER2 overexpression (-)

Time (h)	EV				WT				V104L				V104M			
	BG	0.1 nM	1 nM	10 nM	BG	0.1 nM	1 nM	10 nM	BG	0.1 nM	1 nM	10 nM	BG	0.1 nM	1 nM	10 nM
0	0.963	0.821	0.774	0.962	1.383	1.706	2.072	5.270	0.958	0.904	1.086	2.908	1.252	1.078	1.908	4.905
0.5	1.351	1.373	1.490	1.425	2.166	3.221	5.537	7.705	1.342	1.491	4.242	6.745	1.857	1.842	4.828	7.034
1	1.789	2.527	1.893	2.564	3.511	5.606	8.103	10.256	1.729	2.589	6.740	8.816	3.064	3.574	6.559	8.829
1.5	2.763	2.992	3.216	3.553	4.993	7.352	10.198	11.799	2.912	3.812	8.005	9.422	3.912	5.111	8.121	11.066
2	3.108	3.394	3.171	3.807	5.788	8.002	10.403	13.029	3.457	4.850	9.185	10.221	4.502	6.567	9.704	11.289
2.5	2.890	3.848	3.695	4.591	7.148	8.603	9.885	11.452	3.720	5.543	9.283	10.510	5.200	6.296	8.487	11.821
3	3.950	4.093	3.513	4.271	6.652	9.976	10.306	13.780	4.148	6.086	9.851	10.308	5.543	6.782	9.067	12.830
3.5	4.154	4.024	3.993	5.022	7.199	10.533	12.427	13.059	4.062	6.561	10.202	10.850	6.308	8.103	11.909	13.716
4	4.387	4.364	3.699	4.858	7.324	10.070	11.855	13.965	4.843	6.978	9.630	11.009	5.218	8.101	10.522	12.697
4.5	3.891	4.560	4.105	4.828	7.380	10.432	11.168	13.401	4.675	6.396	10.319	11.277	6.172	7.560	10.866	12.810
5	3.598	4.287	4.413	5.347	7.603	10.682	12.505	14.686	4.763	6.924	10.889	11.370	6.805	9.269	11.272	13.799
5.5	4.059	3.960	3.873	4.815	7.744	10.336	13.233	14.337	4.708	7.252	11.680	11.104	6.617	8.611	11.150	12.477
6	3.927	4.785	4.204	5.025	7.521	10.279	12.454	15.002	4.356	6.978	12.222	10.914	6.410	8.820	11.823	13.315
6.5	3.978	4.194	3.887	4.969	7.426	11.519	11.753	14.055	4.773	7.209	11.113	12.669	6.536	9.146	11.979	14.093
7	3.195	4.447	4.382	4.940	7.338	10.794	12.891	14.684	4.364	7.270	11.716	13.171	5.628	8.208	12.072	14.819
7.5	3.140	3.966	3.997	5.362	7.914	10.392	13.068	14.440	4.109	7.164	12.041	13.245	6.055	8.499	11.304	13.805
8	4.103	3.679	3.943	4.906	6.786	10.482	12.729	14.922	4.157	7.086	11.628	13.094	6.114	8.099	10.429	12.749

8.5	3.440	3.877	4.014	4.262	6.229	11.039	10.817	13.808	4.889	7.150	9.949	11.403	6.057	8.867	11.672	13.370
9	3.636	3.806	3.436	4.798	6.449	10.196	12.085	13.003	4.804	6.509	10.876	11.401	5.859	7.857	10.365	13.434
9.5	3.744	3.996	3.646	4.349	6.670	10.048	11.145	14.313	4.697	6.793	12.413	11.746	5.639	7.785	11.704	13.261
10	2.868	3.688	3.685	4.595	5.681	9.061	12.926	13.451	4.535	6.875	11.510	10.009	5.850	7.146	9.076	13.212
10.5	2.727	3.151	3.375	4.704	5.468	8.816	13.049	14.428	3.884	6.444	10.555	12.286	6.046	7.712	11.442	14.079
11	2.772	3.139	3.493	4.026	6.192	9.642	13.067	13.287	4.193	6.647	10.742	11.919	5.535	7.210	11.057	11.411
11.5	3.061	2.892	3.630	3.545	5.368	8.863	11.365	13.876	3.621	7.390	11.196	11.842	4.688	7.074	10.364	12.706
12	2.485	3.312	3.481	4.246	5.592	9.318	13.345	14.952	3.929	6.897	12.193	12.798	5.650	7.427	11.221	13.989

Time (h)	A232V				P262H				G284R				D297Y			
	BG	0.1 nM	1 nM	10 nM	BG	0.1 nM	1 nM	10 nM	BG	0.1 nM	1 nM	10 nM	BG	0.1 nM	1 nM	10 nM
0	1.265	1.063	1.341	2.881	0.957	1.232	2.073	4.933	0.973	1.099	1.226	2.799	1.148	1.388	2.600	5.670
0.5	1.797	1.652	4.921	6.349	1.606	1.948	5.421	7.192	1.567	1.292	4.531	5.578	1.678	2.348	5.813	7.049
1	2.959	2.916	7.293	7.370	2.779	3.591	7.162	8.481	2.410	2.115	7.295	6.473	3.096	3.811	6.877	8.962
1.5	3.510	4.544	8.582	9.254	3.213	5.195	9.349	9.625	2.515	3.824	8.047	8.693	3.618	5.392	8.585	10.057
2	4.288	5.364	10.456	9.931	3.851	6.015	9.806	10.429	3.843	4.361	8.420	8.543	3.894	5.421	9.116	10.609
2.5	3.901	6.037	8.802	9.064	4.536	6.041	9.610	12.013	3.723	5.093	9.484	9.389	4.761	6.601	9.279	9.836
3	4.525	6.401	9.072	10.465	4.540	6.618	10.671	12.644	3.858	5.656	9.486	9.171	4.582	7.125	10.029	11.341
3.5	4.549	6.038	9.085	10.585	5.008	6.627	10.050	12.351	4.514	6.405	9.010	9.414	5.381	7.441	10.476	11.412
4	5.852	6.569	9.819	10.526	5.067	6.739	10.854	13.937	4.200	6.453	10.126	10.041	5.532	7.266	9.969	11.446
4.5	5.147	6.796	9.604	10.499	4.623	7.353	9.824	13.933	4.062	6.115	9.668	9.861	5.301	7.963	9.934	11.280
5	5.350	6.996	10.987	11.092	4.365	8.495	10.379	14.058	5.105	5.767	10.315	10.512	5.584	8.340	10.627	11.972

5.5	4.815	7.127	11.253	10.454	4.230	7.804	11.240	14.225	4.671	6.324	10.698	9.975	5.421	7.720	10.373	12.110
6	5.523	5.772	10.476	11.006	5.015	8.289	10.001	13.945	4.291	6.446	10.284	10.908	5.056	7.505	10.131	12.283
6.5	4.840	7.030	9.890	10.237	5.427	8.745	11.324	13.876	4.921	6.621	10.464	12.069	5.534	9.256	11.842	12.473
7	5.750	7.272	10.142	11.742	4.270	8.187	12.552	14.565	4.425	6.502	9.986	11.386	5.336	7.685	10.777	13.360
7.5	5.317	7.337	10.710	10.958	5.035	7.852	11.272	14.940	4.722	6.834	11.254	9.863	5.025	6.992	10.715	12.972
8	4.828	7.139	10.939	11.025	4.810	8.250	10.990	13.222	3.717	5.830	9.412	11.530	4.862	8.267	11.421	12.243
8.5	4.460	6.007	10.419	10.673	5.046	8.143	10.179	13.160	4.094	5.874	10.465	9.346	4.258	7.322	11.779	12.011
9	4.783	5.545	10.486	10.529	5.143	7.443	10.503	13.330	3.980	5.594	11.179	10.620	4.461	7.776	11.392	12.681
9.5	4.445	6.441	9.429	12.488	4.869	8.239	11.599	12.017	4.242	6.442	11.484	10.688	4.841	7.419	10.997	11.768
10	4.124	6.362	9.243	11.375	3.958	8.260	9.958	13.223	3.184	5.598	11.751	11.241	4.569	7.380	10.543	11.744
10.5	4.088	6.051	10.568	11.574	4.576	7.713	10.578	13.381	3.821	5.747	10.619	11.816	4.748	6.888	11.110	12.579
11	4.577	6.147	10.803	13.129	4.897	7.922	10.989	13.486	3.294	5.268	11.428	10.304	4.567	7.038	10.332	12.870
11.5	3.650	4.748	10.493	12.466	3.646	7.521	10.601	13.241	3.789	6.080	11.710	11.485	4.153	6.893	10.548	11.651
12	4.298	6.027	11.169	12.073	4.463	6.792	11.039	13.456	3.665	5.544	10.847	11.977	4.591	7.042	11.208	12.735

Time (h)	G325R				T355I				S846I				E928G			
	BG	0.1 nM	1 nM	10 nM	BG	0.1 nM	1 nM	10 nM	BG	0.1 nM	1 nM	10 nM	BG	0.1 nM	1 nM	10 nM
0	1.147	1.244	1.387	3.381	1.800	1.270	2.645	5.466	1.319	0.660	1.011	3.215	0.902	0.965	2.137	5.428
0.5	1.930	1.735	4.718	7.130	2.650	1.885	5.956	8.076	1.989	1.072	4.149	6.429	1.238	1.866	5.222	6.689
1	2.605	3.315	7.490	8.349	3.403	3.468	7.788	9.425	2.869	1.850	6.189	7.187	2.263	3.586	7.186	8.574
1.5	3.511	4.882	8.523	9.012	4.786	5.711	9.094	10.208	3.903	4.036	6.846	8.598	3.199	4.788	8.369	9.601
2	3.737	4.785	8.208	9.260	5.438	6.155	9.403	9.876	4.676	4.401	7.415	9.046	3.113	5.484	8.856	9.947

2.5	4.448	4.781	8.512	9.833	5.684	6.156	9.437	10.342	5.208	4.247	8.240	9.175	3.927	5.354	8.655	9.996
3	5.055	7.356	9.293	10.597	5.618	6.753	10.095	10.384	5.624	5.107	8.182	9.085	3.658	6.467	8.887	10.948
3.5	4.776	6.496	8.987	11.065	5.982	6.619	10.383	12.407	5.541	5.000	8.645	9.937	4.615	7.404	10.010	10.291
4	5.442	7.275	9.356	11.218	5.448	7.726	11.223	11.378	5.615	5.143	9.301	10.056	4.461	7.180	10.926	11.402
4.5	5.127	6.460	8.755	11.801	6.231	8.150	10.326	11.285	5.649	5.724	9.316	9.241	5.029	6.217	9.348	11.405
5	4.872	6.357	10.328	12.343	4.971	7.855	11.175	13.154	5.096	6.058	9.643	10.134	4.623	7.103	8.986	10.949
5.5	4.771	7.267	10.131	11.529	6.816	7.669	10.806	12.885	6.007	5.875	9.937	10.917	3.992	6.732	9.993	10.899
6	4.252	6.950	10.517	12.311	6.241	7.204	10.508	12.962	5.139	6.108	10.141	10.986	5.113	7.493	9.889	11.278
6.5	4.593	5.957	10.678	12.801	5.856	8.901	12.106	13.846	4.733	6.101	10.549	10.651	5.335	7.149	9.311	13.131
7	4.728	6.398	10.431	12.615	6.191	8.019	12.154	14.191	5.691	6.660	10.774	12.065	4.518	7.460	11.104	12.424
7.5	4.822	6.346	10.704	12.527	5.578	7.096	10.734	12.438	5.660	5.527	11.871	11.467	4.393	7.275	9.991	11.771
8	4.559	6.452	10.381	12.279	5.762	6.905	10.199	13.258	5.330	5.951	10.861	10.997	4.776	5.951	9.731	11.495
8.5	5.056	6.838	9.729	12.167	5.972	7.215	10.764	12.680	5.075	6.127	10.590	10.826	3.878	6.663	8.908	11.458
9	4.091	6.142	10.121	12.255	5.725	6.349	10.699	12.682	4.511	5.705	10.013	10.906	4.334	5.478	8.991	11.103
9.5	4.548	6.720	11.019	12.424	5.807	6.704	11.331	11.691	4.883	5.210	10.440	11.521	3.805	6.649	9.113	10.002
10	4.674	5.635	10.529	12.915	5.987	6.929	11.518	12.925	4.797	5.218	10.547	11.136	3.785	5.551	9.821	10.918
10.5	4.144	6.534	11.809	12.868	5.241	6.482	10.033	12.337	5.130	5.192	9.665	9.786	3.599	6.859	8.603	10.917
11	3.715	5.526	11.251	12.384	4.908	6.976	10.716	11.146	4.656	5.510	8.816	10.663	3.963	6.166	10.173	10.887
11.5	3.892	5.712	10.595	12.990	4.749	6.456	11.601	11.309	4.717	5.663	9.729	10.554	3.671	6.521	9.183	12.367
12	3.964	5.985	10.772	12.294	5.043	6.419	10.544	12.004	4.797	6.031	9.543	11.134	4.221	7.394	9.227	13.041

B) HER2 overexpression (+)

Time (h)	EV				WT				V104L				V104M			
	BG	0.1 nM	1 nM	10 nM	BG	0.1 nM	1 nM	10 nM	BG	0.1 nM	1 nM	10 nM	BG	0.1 nM	1 nM	10 nM
0	0.479	0.538	0.709	0.665	0.633	0.657	0.970	4.378	0.614	0.580	0.767	1.036	0.856	0.940	1.360	3.774
0.5	0.991	1.030	1.239	1.209	1.267	1.495	4.081	6.838	1.096	1.360	2.957	4.879	1.846	2.099	5.436	6.289
1	1.633	1.688	1.734	2.063	2.249	2.727	5.799	7.322	1.864	2.414	5.370	7.358	2.621	3.690	7.541	8.475
1.5	1.865	2.400	3.106	2.684	2.962	4.372	7.469	9.065	2.700	3.852	7.542	7.503	4.219	6.147	8.555	9.480
2	2.986	3.105	4.084	3.374	4.017	5.035	9.477	9.433	3.615	5.085	8.587	8.833	4.157	7.827	10.541	7.870
2.5	3.640	3.414	4.379	4.371	4.690	5.545	9.148	11.032	3.186	5.871	9.582	8.837	4.954	7.277	10.333	10.035
3	3.316	3.965	4.076	4.405	4.797	5.634	8.626	9.932	3.748	5.758	10.036	9.191	5.497	8.583	9.723	10.631
3.5	3.751	3.706	4.652	5.049	5.014	5.727	9.592	9.718	4.697	5.814	8.967	9.629	5.785	8.127	10.108	10.438
4	3.500	3.756	4.589	5.064	4.952	7.013	10.538	11.199	5.160	6.804	9.969	10.024	6.033	8.731	10.076	10.831
4.5	3.313	4.348	4.037	4.804	4.863	6.443	10.664	10.699	5.023	6.099	10.799	11.401	6.471	7.960	9.127	10.080
5	3.094	4.085	3.943	4.861	4.834	6.106	10.633	11.545	4.653	6.775	9.620	11.042	6.399	8.056	10.210	10.595
5.5	3.357	3.435	4.657	4.677	5.033	6.915	10.609	9.859	5.143	6.186	10.689	11.391	6.423	8.493	10.683	10.217
6	3.616	4.124	3.877	4.114	4.605	6.388	10.142	12.277	5.123	6.321	10.559	10.591	6.097	8.565	11.682	10.579
6.5	3.345	3.724	3.739	3.988	4.743	7.434	10.347	12.581	5.005	6.550	9.763	10.436	5.126	8.241	10.551	11.589
7	3.627	3.794	3.452	4.558	4.616	7.263	9.707	10.944	5.207	6.731	10.036	10.956	6.100	7.775	10.481	11.172
7.5	3.175	3.682	3.125	4.176	4.570	6.381	9.647	11.193	3.893	6.265	10.417	11.162	5.673	7.669	9.719	11.749
8	3.088	3.238	3.570	4.339	5.105	7.150	10.396	11.876	4.027	5.925	11.387	10.687	5.692	8.732	9.918	10.882
8.5	3.031	3.357	3.058	4.066	4.441	6.971	8.948	11.933	3.771	6.142	11.799	12.271	5.402	8.198	9.667	11.170
9	2.509	2.855	3.043	3.730	4.543	7.273	8.904	11.567	3.858	5.319	11.504	10.607	5.467	7.697	9.332	11.067
9.5	2.662	2.585	3.410	4.138	4.583	7.032	8.647	10.306	3.866	5.401	11.361	11.741	5.264	8.033	9.522	10.892

10	2.775	2.450	2.787	4.126	4.189	6.714	9.186	11.791	4.400	5.282	10.560	9.822	5.470	7.682	9.501	10.565
10.5	2.759	2.573	2.778	3.357	4.232	6.129	8.459	11.071	3.527	4.564	10.730	10.251	5.552	6.399	10.152	10.679
11	2.071	2.583	1.886	3.397	4.136	6.750	9.036	10.084	3.503	5.508	10.431	10.646	5.683	6.897	10.953	10.318
11.5	2.083	2.815	2.798	3.450	4.269	4.917	9.447	11.891	4.363	5.308	11.018	10.692	6.119	6.828	9.441	13.054
12	2.416	3.058	2.811	3.168	4.365	6.931	9.902	11.955	4.804	5.682	11.717	10.102	6.053	8.771	11.784	12.047

Time (h)	A232V				P262H				G284R				D297Y			
	BG	0.1 nM	1 nM	10 nM	BG	0.1 nM	1 nM	10 nM	BG	0.1 nM	1 nM	10 nM	BG	0.1 nM	1 nM	10 nM
0	0.987	0.769	0.380	1.305	0.938	1.053	1.333	4.865	0.720	0.608	0.678	1.562	0.742	0.833	1.062	4.796
0.5	1.451	1.157	3.326	6.339	1.684	1.581	5.113	7.436	1.067	1.042	2.969	6.615	1.452	1.691	4.359	6.480
1	2.396	2.128	5.530	7.828	2.710	4.004	8.045	10.040	2.509	2.053	6.810	7.854	2.270	3.290	6.559	9.158
1.5	3.162	3.863	6.946	9.649	3.856	6.091	9.037	9.860	3.157	3.794	7.522	8.767	3.460	5.128	8.109	9.888
2	4.213	4.564	7.822	9.753	3.932	7.898	12.124	10.299	4.242	5.327	9.129	9.045	3.808	4.821	10.059	9.863
2.5	4.818	5.896	9.597	11.332	5.987	8.500	13.731	13.291	4.987	5.797	10.554	9.851	4.413	6.177	9.349	10.583
3	5.593	6.143	8.624	11.088	6.038	9.499	12.668	13.886	4.469	6.040	10.042	9.292	4.886	7.426	8.915	10.840
3.5	5.199	6.412	9.389	12.342	6.667	9.238	13.674	11.591	4.387	6.289	10.173	11.290	5.026	6.948	9.865	10.258
4	5.409	6.995	9.610	12.357	6.135	9.910	12.210	14.172	4.646	6.426	10.532	9.937	5.128	6.861	9.494	10.120
4.5	5.270	6.485	9.640	12.280	5.950	9.191	12.099	14.135	4.939	7.073	11.628	10.929	5.233	6.537	9.555	9.684
5	6.104	7.037	9.669	10.767	6.395	9.034	12.429	12.773	4.430	7.864	11.889	12.099	5.003	7.417	10.229	10.777
5.5	6.566	8.118	10.305	12.491	7.244	9.018	13.898	12.821	5.062	8.041	11.358	11.926	5.474	7.609	10.118	10.862
6	5.241	7.957	11.148	12.696	5.759	9.976	12.909	13.642	5.484	7.144	11.530	11.549	4.737	7.250	9.674	11.397
6.5	6.083	7.229	11.228	11.915	6.004	10.146	12.810	13.792	4.897	6.991	11.909	12.530	4.622	7.302	10.693	10.146

7	6.607	7.033	11.459	11.548	5.848	8.134	13.145	14.248	5.235	6.817	11.588	11.250	4.481	7.355	10.715	11.190
7.5	6.654	7.022	10.718	12.390	6.640	8.965	14.325	13.373	4.010	6.507	10.692	12.494	5.151	7.673	10.553	11.299
8	5.516	5.880	9.166	11.998	5.855	7.628	15.586	12.590	4.141	7.243	10.613	10.545	4.202	7.399	9.483	9.869
8.5	4.828	6.114	11.719	12.274	5.527	8.089	14.502	13.774	5.154	6.583	10.278	11.541	4.996	6.695	9.683	11.981
9	5.027	6.408	10.561	11.287	5.944	7.081	15.856	13.766	4.371	5.875	9.757	11.190	4.913	5.274	10.509	9.793
9.5	5.227	6.717	9.994	11.662	5.792	7.155	15.225	12.830	3.777	6.558	9.616	11.899	4.441	6.624	10.769	11.633
10	5.488	6.521	11.317	10.698	5.665	7.855	14.126	13.313	4.585	6.856	10.822	11.682	4.308	7.401	10.104	12.848
10.5	5.295	5.681	9.591	11.684	4.646	7.169	14.718	13.064	3.968	6.830	10.033	10.951	4.519	5.862	9.551	12.102
11	4.983	7.144	10.674	12.404	5.227	8.623	13.041	11.593	4.096	5.751	10.281	11.697	3.993	5.894	10.408	13.851
11.5	4.262	6.106	10.435	12.135	5.522	7.142	14.399	15.638	2.895	6.025	10.855	11.865	3.597	6.697	11.816	12.357
12	5.465	5.853	11.383	12.090	4.889	8.686	14.548	12.540	3.648	6.227	10.113	12.366	4.062	6.235	10.707	12.850

Time (h)	G325R				T355I				Q809R				S846I			
	BG	0.1 nM	1 nM	10 nM	BG	0.1 nM	1 nM	10 nM	BG	0.1 nM	1 nM	10 nM	BG	0.1 nM	1 nM	10 nM
0	0.565	0.803	0.861	1.430	0.691	0.894	1.842	4.814	1.293	1.626	1.228	3.009	0.819	0.669	0.802	1.782
0.5	0.944	1.339	4.141	6.290	1.599	1.652	5.099	6.901	2.056	1.801	5.342	7.746	1.401	1.181	4.177	6.345
1	1.537	2.321	5.993	8.039	2.144	3.427	6.403	7.889	2.621	4.808	7.600	8.271	2.059	2.301	6.704	7.720
1.5	2.095	3.839	8.350	8.123	2.933	4.337	8.386	9.630	4.341	6.953	10.980	9.238	2.532	3.585	8.206	8.701
2	3.476	4.940	8.080	10.184	3.997	5.472	8.854	9.530	5.785	8.617	12.100	11.797	3.909	5.161	8.737	8.968
2.5	3.232	5.813	9.328	10.158	4.919	6.199	8.749	10.773	6.625	9.997	13.954	11.107	4.062	4.996	9.366	9.598
3	4.354	7.716	9.806	11.207	4.593	7.046	9.466	10.793	8.429	11.227	15.097	12.643	4.280	6.559	8.791	10.354
3.5	4.850	7.057	9.559	11.597	5.545	7.317	9.841	11.822	7.702	9.927	15.683	14.642	3.868	6.494	10.330	10.674

4	4.485	6.270	9.483	10.365	5.478	7.373	9.721	11.816	7.800	11.538	14.250	14.973	4.498	5.976	10.962	11.069
4.5	4.088	7.262	10.912	11.053	5.652	7.414	11.086	12.173	7.249	10.891	16.352	13.314	5.226	6.595	10.773	10.824
5	4.317	7.199	11.529	10.758	5.540	6.788	9.437	9.674	8.293	9.652	15.621	14.305	5.551	6.294	11.399	11.166
5.5	5.274	6.576	10.360	12.758	6.303	7.554	10.507	11.911	8.919	10.163	15.296	15.459	6.074	6.868	10.456	12.078
6	3.295	6.008	11.376	11.264	6.111	8.323	11.207	12.233	7.442	10.876	11.312	16.128	4.439	7.213	11.499	10.863
6.5	3.548	6.652	12.137	10.185	5.925	8.177	10.195	12.145	7.873	9.533	14.216	12.952	3.286	6.992	10.569	11.239
7	3.643	6.490	10.505	11.060	5.391	7.541	11.652	12.207	6.518	9.383	16.366	12.348	4.348	6.097	11.385	10.324
7.5	2.555	6.517	10.740	10.731	4.422	6.932	10.202	13.191	6.356	10.436	11.687	12.106	4.111	6.003	9.545	11.879
8	3.367	6.730	10.029	10.845	4.619	7.217	10.124	12.585	6.657	9.509	12.904	13.155	4.487	6.068	11.526	11.407
8.5	3.674	6.252	10.256	11.656	4.964	7.699	10.894	13.156	5.076	8.215	12.681	14.228	4.370	6.005	9.903	12.460
9	3.784	5.581	9.236	10.362	4.879	7.022	11.050	11.737	5.649	8.446	16.454	12.735	3.720	5.571	10.227	10.833
9.5	3.405	5.465	10.160	11.791	5.882	7.215	10.008	13.605	4.374	8.220	13.484	12.640	4.088	6.236	10.223	11.459
10	3.275	6.005	9.856	11.264	5.358	6.687	9.883	11.776	4.584	7.231	13.764	13.982	4.408	6.161	9.931	12.265
10.5	2.807	5.408	9.438	12.227	4.598	6.491	10.900	13.160	4.920	7.026	13.589	14.671	3.569	5.394	9.857	11.532
11	3.824	6.803	9.976	12.475	4.003	7.076	10.929	11.397	5.289	8.243	14.133	10.825	4.041	5.818	10.380	12.100
11.5	2.280	6.805	10.165	11.385	4.817	6.963	10.168	13.932	5.675	7.234	13.353	10.264	2.927	5.993	10.714	11.167
12	2.832	5.758	10.272	11.505	4.012	5.825	10.013	11.250	5.318	6.987	16.123	8.948	3.230	6.126	10.231	13.004

Time (h)	E928G				EV (HER2-)				WT (HER2-)			
	BG	0.1 nM	1 nM	10 nM	BG	0.1 nM	1 nM	10 nM	BG	0.1 nM	1 nM	10 nM
0	0.598	0.707	1.307	4.427	0.737	0.498	0.584	0.601	0.992	0.981	1.486	4.354
0.5	1.256	1.033	4.915	6.974	0.736	1.028	1.046	1.004	1.550	1.913	4.854	6.979
1	2.481	2.744	6.509	6.740	1.542	1.768	1.868	1.716	2.900	3.794	7.237	9.031
1.5	3.142	4.518	7.133	9.648	1.957	2.630	3.210	2.909	4.207	5.207	8.548	9.710
2	3.840	5.668	8.979	10.029	2.238	3.229	3.753	3.052	5.467	6.145	10.006	10.591
2.5	5.103	6.523	9.866	9.749	3.471	3.794	4.824	4.241	6.991	7.471	11.182	12.430
3	5.307	5.537	10.788	9.999	2.736	4.257	4.878	4.085	6.630	8.221	11.697	12.430
3.5	5.634	6.281	12.281	11.976	4.054	3.933	4.057	4.468	6.387	8.604	12.333	11.960
4	5.428	6.738	12.139	12.721	3.593	4.651	4.771	4.857	6.192	9.353	11.364	11.086
4.5	5.185	6.710	11.356	11.890	3.465	4.967	4.976	5.042	6.942	9.242	10.683	12.222
5	5.851	7.963	11.553	10.894	2.776	5.180	5.250	5.051	6.907	9.916	12.112	12.911
5.5	6.510	7.776	11.885	13.155	3.714	5.161	5.018	4.048	5.894	10.496	11.716	13.094
6	6.682	7.130	12.164	11.815	3.039	4.460	5.342	4.717	6.454	8.185	12.641	13.415
6.5	4.622	7.830	11.204	11.518	2.938	4.929	4.996	4.693	6.340	9.931	11.995	12.547
7	4.645	8.291	12.548	11.981	2.825	4.572	5.226	4.977	6.032	8.917	12.883	13.429
7.5	4.750	7.272	11.565	11.165	2.844	4.923	5.558	4.595	6.197	9.101	11.619	11.961
8	3.860	7.632	12.352	12.355	3.152	3.057	4.245	3.925	5.557	8.785	12.035	12.576
8.5	4.065	7.185	10.950	11.932	2.909	4.763	4.521	4.556	6.066	10.695	12.200	12.467
9	4.037	7.750	11.381	11.561	2.572	4.588	4.502	3.618	5.640	7.694	10.003	11.759
9.5	6.017	7.070	12.887	11.021	2.773	4.010	4.152	3.248	5.903	7.666	11.975	12.367
10	4.348	6.486	12.546	11.495	3.088	2.806	3.325	3.833	6.066	8.648	11.233	12.751

10.5	3.729	6.032	11.700	11.235	2.654	4.173	4.607	3.779	5.647	7.223	10.637	10.406
11	5.209	7.041	11.549	11.706	3.008	3.983	4.350	3.752	5.136	8.616	11.759	13.207
11.5	3.960	6.106	12.574	10.363	2.779	3.160	4.100	3.239	5.513	10.092	11.385	13.447
12	4.346	8.516	11.635	12.153	2.911	3.474	4.450	4.161	7.319	9.395	11.555	11.954
