# Supplementary material for: Patritumab deruxtecan (HER3-DXd), a novel HER3 directed antibody drug conjugate, exhibits in vitro activity against breast cancer cells expressing HER3 mutations with and without HER2 overexpression
Source: PLoS One. 2022 May 3;17(5):e0267027. doi: 10.1371/journal.pone.0267027 (PMC9064083; doi:10.1371/journal.pone.0267027)
Supplement: S3 Table — (PDF) [file pone.0267027.s008.pdf]

A) HER2 overexpression (-)

Time (h)	EV				WT				V104L				V104M			
	BG	0.1 nM	1 nM	10 nM	BG	0.1 nM	1 nM	10 nM	BG	0.1 nM	1 nM	10 nM	BG	0.1 nM	1 nM	10 nM
0	0.000	0.000	0.000	0.000	0.002	0.004	0.000	0.086	0.000	0.000	0.004	0.000	0.000	0.003	0.000	0.104
0.5	0.010	0.003	0.010	0.000	0.013	0.020	0.086	0.262	0.000	0.005	0.056	0.280	0.000	0.003	0.064	0.296
1	0.012	0.029	0.015	0.027	0.048	0.061	0.250	0.468	0.010	0.022	0.227	0.590	0.020	0.020	0.196	0.495
1.5	0.051	0.065	0.060	0.059	0.098	0.171	0.492	0.647	0.035	0.071	0.448	0.611	0.035	0.078	0.380	0.724
2	0.089	0.075	0.084	0.080	0.131	0.214	0.504	0.901	0.057	0.105	0.630	0.783	0.071	0.145	0.542	0.799
2.5	0.076	0.122	0.110	0.138	0.213	0.303	0.500	0.754	0.074	0.138	0.675	0.900	0.097	0.166	0.470	0.925
3	0.119	0.120	0.090	0.117	0.207	0.388	0.576	0.993	0.108	0.161	0.777	0.860	0.119	0.197	0.518	1.014
3.5	0.131	0.143	0.120	0.150	0.234	0.447	0.871	0.861	0.088	0.216	0.783	0.992	0.164	0.311	0.842	1.135
4	0.134	0.154	0.097	0.132	0.228	0.439	0.733	0.994	0.122	0.249	0.808	1.010	0.114	0.300	0.732	1.092
4.5	0.108	0.171	0.105	0.137	0.231	0.470	0.721	0.926	0.122	0.221	0.876	1.043	0.164	0.232	0.787	0.965
5	0.098	0.143	0.157	0.181	0.223	0.455	0.883	1.130	0.126	0.244	0.958	1.049	0.163	0.392	0.812	1.228
5.5	0.117	0.126	0.110	0.138	0.246	0.479	0.929	1.153	0.115	0.259	1.096	0.987	0.160	0.304	0.814	1.003
6	0.111	0.147	0.118	0.155	0.221	0.454	0.905	1.187	0.084	0.266	1.147	0.930	0.142	0.345	0.831	1.094
6.5	0.109	0.125	0.103	0.118	0.203	0.502	0.819	1.105	0.114	0.281	1.006	1.172	0.157	0.355	0.901	1.187
7	0.063	0.137	0.116	0.139	0.198	0.441	0.951	1.126	0.073	0.268	1.141	1.298	0.096	0.312	0.899	1.184
7.5	0.072	0.103	0.083	0.161	0.216	0.404	0.986	1.126	0.071	0.295	1.140	1.287	0.133	0.334	0.771	1.139
8	0.103	0.083	0.099	0.116	0.173	0.472	0.946	1.185	0.055	0.284	1.112	1.276	0.119	0.300	0.777	1.058

8.5	0.071	0.093	0.105	0.085	0.138	0.526	0.669	1.096	0.088	0.315	0.918	0.989	0.129	0.349	0.862	1.059
9	0.065	0.095	0.067	0.119	0.149	0.477	0.772	0.980	0.092	0.272	0.976	1.005	0.113	0.330	0.648	1.064
9.5	0.076	0.098	0.071	0.098	0.164	0.509	0.740	1.085	0.074	0.305	1.266	1.111	0.103	0.326	0.809	1.025
10	0.036	0.091	0.075	0.110	0.102	0.413	0.901	1.088	0.073	0.293	1.070	0.838	0.120	0.281	0.570	1.024
10.5	0.045	0.066	0.068	0.106	0.120	0.421	0.906	1.081	0.050	0.263	1.008	1.112	0.119	0.328	0.845	1.177
11	0.046	0.062	0.063	0.091	0.093	0.411	0.969	1.031	0.071	0.260	1.016	1.112	0.090	0.269	0.805	0.838
11.5	0.051	0.061	0.072	0.068	0.079	0.420	0.726	1.052	0.037	0.320	1.083	1.045	0.059	0.259	0.690	0.954
12	0.034	0.062	0.065	0.104	0.091	0.425	0.997	1.123	0.056	0.302	1.200	1.211	0.097	0.255	0.798	1.125

Time (h)	A232V				P262H				G284R				D297Y			
	BG	0.1 nM	1 nM	10 nM	BG	0.1 nM	1 nM	10 nM	BG	0.1 nM	1 nM	10 nM	BG	0.1 nM	1 nM	10 nM
0	0.000	0.000	0.000	0.000	0.000	0.000	0.000	0.087	0.000	0.000	0.000	0.000	0.000	0.000	0.000	0.097
0.5	0.005	0.002	0.049	0.185	0.001	0.005	0.099	0.324	0.000	0.000	0.054	0.169	0.004	0.013	0.127	0.302
1	0.020	0.029	0.257	0.302	0.022	0.035	0.276	0.584	0.018	0.012	0.259	0.301	0.042	0.041	0.280	0.606
1.5	0.049	0.070	0.447	0.557	0.043	0.107	0.549	0.764	0.015	0.045	0.399	0.561	0.050	0.131	0.521	0.789
2	0.085	0.121	0.681	0.688	0.056	0.126	0.632	0.879	0.087	0.067	0.464	0.586	0.067	0.118	0.615	0.839
2.5	0.068	0.156	0.483	0.619	0.106	0.154	0.646	1.048	0.063	0.100	0.630	0.764	0.112	0.220	0.667	0.783
3	0.102	0.178	0.511	0.780	0.108	0.204	0.771	1.201	0.083	0.121	0.687	0.752	0.108	0.258	0.793	1.017
3.5	0.106	0.164	0.540	0.843	0.124	0.253	0.752	1.078	0.103	0.185	0.691	0.768	0.151	0.312	0.850	1.045
4	0.148	0.217	0.630	0.788	0.131	0.272	0.863	1.351	0.070	0.203	0.823	0.882	0.151	0.317	0.799	1.087
4.5	0.113	0.246	0.646	0.856	0.103	0.288	0.737	1.363	0.079	0.183	0.819	0.839	0.134	0.350	0.837	0.990
5	0.136	0.258	0.807	0.810	0.091	0.370	0.796	1.337	0.132	0.171	0.912	0.935	0.128	0.377	0.929	1.096

5.5	0.115	0.275	0.917	0.820	0.097	0.358	0.901	1.398	0.106	0.205	0.953	0.845	0.114	0.364	0.870	1.194
6	0.120	0.158	0.817	0.899	0.104	0.366	0.772	1.308	0.080	0.216	0.901	0.942	0.104	0.346	0.808	1.170
6.5	0.081	0.288	0.775	0.810	0.114	0.393	0.953	1.320	0.095	0.244	0.913	1.129	0.130	0.490	1.138	1.163
7	0.115	0.313	0.706	0.974	0.076	0.351	1.161	1.442	0.089	0.229	0.851	1.063	0.122	0.398	0.939	1.312
7.5	0.113	0.312	0.767	0.886	0.100	0.325	0.952	1.466	0.083	0.240	1.042	0.787	0.095	0.339	0.966	1.294
8	0.072	0.308	0.800	0.918	0.073	0.397	0.954	1.248	0.050	0.224	0.796	1.076	0.090	0.417	1.043	1.163
8.5	0.049	0.216	0.807	0.850	0.079	0.375	0.767	1.223	0.072	0.219	0.933	0.752	0.060	0.337	1.109	1.071
9	0.081	0.187	0.831	0.828	0.096	0.307	0.902	1.206	0.062	0.214	1.037	0.892	0.074	0.376	1.067	1.176
9.5	0.056	0.269	0.700	1.075	0.079	0.382	1.084	1.054	0.060	0.249	1.094	0.946	0.088	0.370	0.924	1.106
10	0.052	0.261	0.663	0.927	0.042	0.381	0.894	1.292	0.049	0.223	1.167	0.991	0.066	0.357	0.912	1.099
10.5	0.057	0.251	0.882	0.950	0.065	0.347	0.922	1.233	0.064	0.223	1.008	1.155	0.054	0.293	1.007	1.151
11	0.065	0.253	0.852	1.154	0.086	0.363	0.937	1.316	0.032	0.177	1.101	0.942	0.062	0.350	0.930	1.241
11.5	0.042	0.165	0.888	1.029	0.055	0.340	0.976	1.272	0.062	0.250	1.217	1.035	0.065	0.326	0.989	1.033
12	0.057	0.225	0.952	1.014	0.081	0.301	1.013	1.240	0.057	0.219	1.001	1.175	0.080	0.363	1.018	1.218

Time (h)	G325R				T355I				S846I				E928G			
	BG	0.1 nM	1 nM	10 nM	BG	0.1 nM	1 nM	10 nM	BG	0.1 nM	1 nM	10 nM	BG	0.1 nM	1 nM	10 nM
0	0.001	0.004	0.000	0.000	0.005	0.002	0.000	0.131	0.000	0.000	0.000	0.000	0.000	0.000	0.000	0.148
0.5	0.009	0.000	0.049	0.234	0.028	0.007	0.152	0.465	0.013	0.000	0.039	0.269	0.001	0.007	0.123	0.344
1	0.022	0.020	0.238	0.460	0.052	0.040	0.298	0.652	0.019	0.005	0.187	0.363	0.016	0.042	0.297	0.590
1.5	0.055	0.071	0.428	0.620	0.116	0.092	0.546	0.809	0.063	0.045	0.317	0.600	0.054	0.084	0.455	0.777
2	0.076	0.065	0.464	0.595	0.145	0.150	0.594	0.746	0.107	0.071	0.423	0.701	0.064	0.135	0.532	0.802

2.5	0.090	0.099	0.547	0.791	0.162	0.138	0.679	0.853	0.150	0.090	0.549	0.762	0.087	0.151	0.568	0.853
3	0.143	0.214	0.644	0.830	0.168	0.210	0.743	0.824	0.182	0.112	0.568	0.740	0.081	0.224	0.641	0.965
3.5	0.110	0.204	0.601	0.921	0.177	0.206	0.751	1.121	0.170	0.116	0.686	0.839	0.136	0.329	0.799	0.956
4	0.142	0.246	0.672	0.991	0.137	0.299	0.914	1.040	0.184	0.119	0.765	0.804	0.113	0.318	0.977	1.120
4.5	0.134	0.245	0.597	1.114	0.173	0.342	0.835	1.014	0.165	0.210	0.830	0.780	0.132	0.247	0.803	1.083
5	0.098	0.231	0.818	1.163	0.111	0.310	0.955	1.259	0.159	0.223	0.811	0.873	0.109	0.327	0.762	1.066
5.5	0.097	0.294	0.772	0.961	0.196	0.320	0.898	1.186	0.211	0.200	0.900	0.975	0.093	0.295	0.850	1.002
6	0.069	0.255	0.834	1.030	0.186	0.289	0.804	1.186	0.155	0.233	0.893	0.951	0.129	0.352	0.880	1.086
6.5	0.069	0.227	0.856	1.121	0.139	0.411	1.045	1.356	0.119	0.247	0.922	0.873	0.147	0.349	0.815	1.304
7	0.086	0.284	0.877	1.145	0.162	0.344	1.035	1.396	0.168	0.275	0.977	1.087	0.088	0.401	1.086	1.235
7.5	0.097	0.256	0.844	1.065	0.126	0.262	0.853	1.149	0.153	0.192	1.099	0.970	0.096	0.386	0.899	1.126
8	0.065	0.263	0.824	1.100	0.129	0.294	0.806	1.315	0.156	0.229	1.018	0.943	0.094	0.282	0.846	1.103
8.5	0.091	0.272	0.721	1.063	0.145	0.284	0.855	1.086	0.125	0.248	0.944	0.905	0.066	0.325	0.684	1.084
9	0.070	0.251	0.835	1.114	0.132	0.225	0.850	1.147	0.104	0.197	0.843	0.999	0.092	0.244	0.702	1.011
9.5	0.080	0.277	0.895	1.206	0.121	0.294	0.986	1.064	0.113	0.182	0.923	1.049	0.078	0.309	0.766	0.884
10	0.070	0.212	0.826	1.212	0.148	0.281	0.972	1.159	0.102	0.180	0.917	0.958	0.062	0.242	0.821	1.009
10.5	0.057	0.313	1.022	1.167	0.115	0.227	0.745	1.046	0.118	0.202	0.815	0.809	0.051	0.338	0.682	1.059
11	0.039	0.220	0.950	1.095	0.096	0.284	0.878	0.926	0.085	0.231	0.725	0.937	0.047	0.284	0.938	1.007
11.5	0.046	0.233	0.820	1.191	0.080	0.245	0.988	0.951	0.103	0.227	0.856	0.963	0.055	0.301	0.750	1.225
12	0.060	0.268	0.851	1.134	0.103	0.255	0.838	1.042	0.111	0.255	0.826	0.998	0.066	0.384	0.785	1.256

B) HER2 overexpression (+)

Time (h)	EV				WT				V104L				V104M			
	BG	0.1 nM	1 nM	10 nM	BG	0.1 nM	1 nM	10 nM	BG	0.1 nM	1 nM	10 nM	BG	0.1 nM	1 nM	10 nM
0	0.001	0.000	0.005	0.000	0.000	0.004	0.000	0.090	0.000	0.003	0.000	0.000	0.000	0.004	0.000	0.032
0.5	0.002	0.005	0.002	0.000	0.006	0.003	0.065	0.395	0.010	0.011	0.006	0.145	0.008	0.011	0.076	0.232
1	0.021	0.016	0.022	0.018	0.037	0.028	0.211	0.501	0.014	0.028	0.150	0.397	0.039	0.051	0.224	0.429
1.5	0.024	0.049	0.053	0.042	0.063	0.097	0.412	0.745	0.048	0.072	0.371	0.410	0.107	0.151	0.425	0.623
2	0.065	0.076	0.105	0.072	0.106	0.135	0.657	0.781	0.090	0.133	0.551	0.666	0.098	0.278	0.607	0.439
2.5	0.094	0.091	0.109	0.099	0.185	0.179	0.707	1.045	0.074	0.181	0.704	0.713	0.175	0.259	0.595	0.728
3	0.075	0.121	0.098	0.101	0.187	0.224	0.648	1.004	0.082	0.202	0.848	0.742	0.216	0.385	0.654	0.796
3.5	0.099	0.087	0.128	0.139	0.184	0.212	0.804	0.904	0.131	0.211	0.708	0.876	0.230	0.410	0.758	0.833
4	0.095	0.098	0.117	0.148	0.171	0.297	0.905	1.164	0.152	0.282	0.813	0.918	0.243	0.438	0.760	0.884
4.5	0.083	0.135	0.103	0.125	0.169	0.276	0.975	1.041	0.134	0.233	0.982	1.096	0.233	0.346	0.729	0.776
5	0.071	0.117	0.070	0.152	0.189	0.287	0.960	1.172	0.122	0.269	0.882	1.042	0.228	0.397	0.862	0.855
5.5	0.079	0.090	0.139	0.097	0.190	0.337	0.918	0.988	0.131	0.251	0.991	1.170	0.221	0.435	0.883	0.840
6	0.083	0.113	0.095	0.086	0.160	0.278	0.911	1.224	0.122	0.278	0.914	0.917	0.202	0.429	0.999	0.848
6.5	0.073	0.093	0.076	0.092	0.149	0.390	0.921	1.307	0.141	0.278	0.890	0.969	0.159	0.414	0.882	0.939
7	0.065	0.088	0.068	0.117	0.145	0.369	0.879	1.062	0.120	0.336	0.974	1.034	0.186	0.361	0.878	0.960
7.5	0.049	0.093	0.072	0.082	0.124	0.343	0.872	1.152	0.056	0.281	0.996	1.049	0.187	0.390	0.784	0.918
8	0.047	0.050	0.081	0.088	0.155	0.348	0.999	1.238	0.083	0.267	1.080	0.959	0.189	0.435	0.793	0.838
8.5	0.053	0.068	0.052	0.078	0.125	0.367	0.718	1.255	0.074	0.241	1.200	1.313	0.154	0.388	0.686	0.931
9	0.052	0.054	0.047	0.067	0.128	0.387	0.793	1.204	0.083	0.204	1.136	1.014	0.159	0.403	0.724	1.001
9.5	0.051	0.047	0.068	0.075	0.112	0.379	0.727	1.018	0.062	0.235	1.087	1.204	0.145	0.448	0.789	0.917

10	0.051	0.053	0.045	0.061	0.105	0.392	0.850	1.217	0.116	0.213	1.011	0.896	0.143	0.390	0.794	0.935
10.5	0.045	0.050	0.044	0.051	0.107	0.308	0.710	1.121	0.074	0.173	0.997	0.968	0.141	0.304	0.864	0.881
11	0.027	0.048	0.031	0.041	0.087	0.356	0.759	1.002	0.071	0.247	0.972	0.974	0.156	0.325	0.963	0.776
11.5	0.029	0.046	0.052	0.049	0.084	0.210	0.892	1.282	0.088	0.250	1.082	1.004	0.174	0.332	0.788	1.161
12	0.043	0.062	0.048	0.047	0.107	0.336	0.917	1.283	0.110	0.276	1.110	0.838	0.153	0.536	0.976	1.028

Time (h)	A232V				P262H				G284R				D297Y			
	BG	0.1 nM	1 nM	10 nM	BG	0.1 nM	1 nM	10 nM	BG	0.1 nM	1 nM	10 nM	BG	0.1 nM	1 nM	10 nM
0	0.000	0.002	0.000	0.000	0.001	0.002	0.000	0.080	0.003	0.002	0.000	0.000	0.007	0.003	0.000	0.069
0.5	0.010	0.000	0.027	0.184	0.002	0.010	0.116	0.346	0.007	0.008	0.027	0.288	0.010	0.008	0.093	0.326
1	0.031	0.005	0.174	0.390	0.009	0.038	0.352	0.623	0.042	0.011	0.246	0.541	0.042	0.045	0.254	0.666
1.5	0.059	0.070	0.313	0.675	0.062	0.136	0.482	0.609	0.062	0.054	0.395	0.643	0.076	0.117	0.500	0.833
2	0.106	0.115	0.420	0.722	0.070	0.243	0.872	0.701	0.095	0.124	0.611	0.669	0.112	0.135	0.751	0.820
2.5	0.169	0.172	0.639	0.949	0.160	0.301	1.128	1.181	0.183	0.160	0.849	0.878	0.149	0.237	0.745	1.028
3	0.198	0.214	0.590	0.919	0.197	0.412	1.105	1.306	0.139	0.173	0.861	0.802	0.183	0.351	0.741	1.058
3.5	0.177	0.263	0.774	1.121	0.233	0.398	1.155	0.960	0.122	0.219	0.880	1.016	0.190	0.333	0.849	0.943
4	0.168	0.331	0.856	1.193	0.186	0.481	1.003	1.382	0.135	0.244	0.969	0.852	0.178	0.334	0.826	1.028
4.5	0.174	0.266	0.820	1.144	0.180	0.441	1.052	1.346	0.155	0.296	1.154	1.089	0.185	0.322	0.907	0.948
5	0.206	0.321	0.805	0.945	0.207	0.438	1.099	1.125	0.136	0.331	1.116	1.188	0.172	0.373	0.981	1.168
5.5	0.231	0.430	0.966	1.196	0.248	0.425	1.375	1.148	0.161	0.384	1.071	1.148	0.190	0.389	0.923	1.155
6	0.146	0.401	1.041	1.240	0.172	0.502	1.176	1.191	0.178	0.289	1.142	1.116	0.167	0.365	0.911	1.252
6.5	0.176	0.335	1.027	1.067	0.166	0.535	1.175	1.286	0.133	0.291	1.173	1.247	0.153	0.359	1.037	0.987

7	0.214	0.351	1.027	1.074	0.159	0.387	1.180	1.322	0.160	0.328	1.103	1.051	0.152	0.366	1.063	1.131
7.5	0.198	0.342	0.956	1.158	0.204	0.444	1.413	1.217	0.081	0.281	1.037	1.325	0.165	0.422	1.084	1.175
8	0.142	0.235	0.712	1.102	0.166	0.336	1.608	1.078	0.084	0.338	0.984	0.965	0.124	0.395	0.829	0.948
8.5	0.119	0.255	1.111	1.141	0.143	0.414	1.465	1.269	0.136	0.260	1.031	1.185	0.137	0.352	0.936	1.269
9	0.110	0.304	0.925	0.994	0.151	0.339	1.540	1.308	0.097	0.261	0.946	1.039	0.114	0.273	1.089	0.936
9.5	0.132	0.359	0.885	1.052	0.134	0.329	1.550	1.177	0.080	0.323	0.970	1.233	0.093	0.346	1.125	1.266
10	0.127	0.322	1.096	0.992	0.139	0.346	1.318	1.251	0.112	0.351	1.144	1.160	0.094	0.417	0.969	1.439
10.5	0.133	0.220	0.820	1.143	0.095	0.343	1.445	1.162	0.092	0.326	1.086	1.121	0.104	0.297	0.919	1.241
11	0.110	0.348	0.969	1.234	0.109	0.427	1.159	0.989	0.088	0.243	1.048	1.268	0.097	0.289	1.073	1.612
11.5	0.089	0.247	0.945	1.144	0.124	0.348	1.437	1.556	0.058	0.284	1.102	1.193	0.074	0.370	1.259	1.292
12	0.144	0.271	1.011	1.093	0.087	0.462	1.362	1.153	0.062	0.289	1.037	1.317	0.085	0.331	1.124	1.429

Time (h)	G325R				T355I				Q809R				S846I			
	BG	0.1 nM	1 nM	10 nM	BG	0.1 nM	1 nM	10 nM	BG	0.1 nM	1 nM	10 nM	BG	0.1 nM	1 nM	10 nM
0	0.000	0.000	0.005	0.000	0.000	0.005	0.000	0.100	0.013	0.011	0.000	0.000	0.003	0.000	0.003	0.001
0.5	0.008	0.006	0.063	0.219	0.008	0.000	0.179	0.344	0.020	0.006	0.108	0.363	0.016	0.000	0.052	0.327
1	0.014	0.009	0.235	0.454	0.025	0.026	0.274	0.566	0.029	0.064	0.268	0.349	0.034	0.014	0.261	0.587
1.5	0.025	0.053	0.514	0.518	0.046	0.072	0.493	0.781	0.061	0.110	0.615	0.513	0.030	0.056	0.547	0.703
2	0.058	0.110	0.516	0.862	0.111	0.144	0.636	0.829	0.119	0.198	0.757	0.830	0.110	0.140	0.714	0.801
2.5	0.056	0.180	0.787	0.820	0.173	0.205	0.668	1.048	0.162	0.298	0.930	0.668	0.127	0.131	0.840	0.946
3	0.101	0.309	0.814	0.985	0.158	0.295	0.815	1.056	0.249	0.424	1.132	0.752	0.132	0.251	0.759	1.043
3.5	0.123	0.330	0.785	1.104	0.200	0.316	0.913	1.230	0.228	0.322	1.319	1.128	0.100	0.248	0.983	1.106

4	0.133	0.251	0.820	0.932	0.181	0.322	0.825	1.233	0.227	0.472	1.186	1.131	0.153	0.277	1.131	1.144
4.5	0.105	0.317	1.032	1.014	0.178	0.307	1.027	1.351	0.199	0.457	1.354	0.920	0.167	0.301	1.105	1.115
5	0.099	0.325	1.177	0.968	0.178	0.324	0.806	0.971	0.252	0.455	1.196	1.123	0.166	0.286	1.147	1.198
5.5	0.136	0.294	0.978	1.311	0.221	0.393	0.938	1.203	0.218	0.458	1.193	1.180	0.209	0.290	1.024	1.343
6	0.070	0.249	1.092	1.082	0.200	0.404	1.070	1.281	0.193	0.517	0.728	1.197	0.117	0.365	1.204	1.114
6.5	0.065	0.300	1.210	0.891	0.159	0.420	0.864	1.281	0.228	0.432	1.041	0.841	0.080	0.356	1.060	1.151
7	0.054	0.306	0.961	1.039	0.172	0.376	1.079	1.214	0.128	0.394	1.293	0.804	0.106	0.276	1.248	1.117
7.5	0.039	0.349	1.001	1.141	0.106	0.345	0.934	1.323	0.090	0.519	0.756	0.791	0.100	0.284	0.941	1.267
8	0.054	0.343	0.948	0.966	0.093	0.329	0.895	1.305	0.129	0.465	0.852	0.996	0.125	0.335	1.199	1.192
8.5	0.069	0.317	0.974	1.116	0.130	0.399	0.920	1.315	0.077	0.351	0.904	1.107	0.095	0.310	1.025	1.393
9	0.064	0.261	0.821	0.945	0.114	0.358	0.969	1.108	0.094	0.381	1.304	0.843	0.068	0.259	1.067	1.158
9.5	0.051	0.271	0.895	1.114	0.173	0.367	0.902	1.420	0.051	0.355	0.945	0.850	0.118	0.322	1.059	1.246
10	0.049	0.307	0.913	1.101	0.125	0.333	0.936	1.217	0.049	0.283	1.040	1.151	0.115	0.306	1.042	1.329
10.5	0.027	0.242	0.876	1.269	0.112	0.326	1.014	1.405	0.081	0.272	0.986	1.141	0.069	0.254	1.013	1.215
11	0.070	0.337	0.958	1.290	0.076	0.360	1.040	1.062	0.093	0.342	1.047	0.653	0.112	0.289	1.068	1.290
11.5	0.024	0.352	1.001	1.137	0.106	0.347	0.940	1.343	0.083	0.289	1.001	0.646	0.055	0.281	1.175	1.128
12	0.046	0.262	1.011	1.112	0.084	0.252	0.860	1.071	0.097	0.265	1.341	0.560	0.051	0.319	1.065	1.406

Time (h)	E928G				EV (HER2-)				WT (HER2-)			
	BG	0.1 nM	1 nM	10 nM	BG	0.1 nM	1 nM	10 nM	BG	0.1 nM	1 nM	10 nM
0	0.001	0.000	0.000	0.080	0.001	0.002	0.000	0.004	0.000	0.000	0.000	0.064
0.5	0.000	0.000	0.158	0.427	0.004	0.009	0.000	0.002	0.013	0.007	0.085	0.317
1	0.034	0.047	0.337	0.380	0.014	0.025	0.019	0.010	0.041	0.039	0.262	0.486
1.5	0.035	0.067	0.407	0.757	0.028	0.053	0.073	0.056	0.080	0.104	0.361	0.538
2	0.063	0.142	0.686	0.843	0.027	0.089	0.100	0.067	0.146	0.149	0.538	0.621
2.5	0.138	0.247	0.709	0.848	0.091	0.122	0.176	0.135	0.201	0.246	0.696	0.807
3	0.169	0.168	0.879	0.865	0.044	0.159	0.188	0.123	0.201	0.278	0.745	0.980
3.5	0.184	0.270	1.118	1.174	0.124	0.131	0.121	0.146	0.186	0.333	0.836	0.883
4	0.151	0.250	1.116	1.186	0.093	0.176	0.191	0.183	0.198	0.388	0.788	0.725
4.5	0.125	0.270	1.081	1.046	0.092	0.179	0.198	0.198	0.194	0.397	0.679	0.909
5	0.170	0.391	1.008	0.959	0.049	0.215	0.235	0.172	0.217	0.420	0.882	1.064
5.5	0.202	0.377	1.068	1.262	0.100	0.213	0.196	0.125	0.168	0.463	0.789	0.983
6	0.190	0.316	1.094	1.023	0.074	0.149	0.206	0.149	0.181	0.294	0.962	1.090
6.5	0.084	0.368	1.041	1.120	0.072	0.192	0.177	0.141	0.168	0.451	0.876	0.964
7	0.101	0.423	1.213	1.118	0.053	0.153	0.186	0.145	0.147	0.319	0.966	1.045
7.5	0.100	0.325	1.048	1.109	0.055	0.181	0.199	0.125	0.160	0.348	0.839	0.819
8	0.086	0.372	1.174	1.193	0.061	0.066	0.142	0.088	0.136	0.309	0.859	0.956
8.5	0.092	0.340	1.000	1.147	0.059	0.149	0.135	0.123	0.142	0.484	0.988	0.988
9	0.085	0.413	1.062	1.095	0.048	0.148	0.125	0.072	0.121	0.255	0.654	0.840
9.5	0.156	0.378	1.219	0.991	0.032	0.108	0.091	0.062	0.131	0.277	0.904	0.947
10	0.096	0.284	1.208	1.099	0.047	0.037	0.060	0.088	0.105	0.312	0.841	0.976

10.5	0.079	0.259	1.082	1.088	0.039	0.101	0.095	0.070	0.093	0.229	0.740	0.723
11	0.142	0.348	1.214	1.035	0.052	0.090	0.097	0.063	0.094	0.336	0.855	0.947
11.5	0.091	0.264	1.210	0.867	0.046	0.053	0.095	0.043	0.108	0.415	0.838	0.985
12	0.098	0.491	1.087	1.076	0.045	0.066	0.091	0.080	0.166	0.380	0.783	0.803
