# Supplementary material for: Patritumab deruxtecan (HER3-DXd), a novel HER3 directed antibody drug conjugate, exhibits in vitro activity against breast cancer cells expressing HER3 mutations with and without HER2 overexpression
Source: PLoS One. 2022 May 3;17(5):e0267027. doi: 10.1371/journal.pone.0267027 (PMC9064083; doi:10.1371/journal.pone.0267027)
Supplement: S4 Table — (PDF) [file pone.0267027.s009.pdf]

A) HER2 overexpression (-)

HER3	Test article	Cell viability (%)					
		#1	#2	#3	#4	Mean	SD
EV	Control	95.2	100.4	100.8	103.7	100.0	3.5
	10 nM HER3-DXd	103.0	107.1	111.3	107.0	107.1	3.4
	10 nM Patritumab	99.7	108.1	116.9	109.0	108.4	7.0
	10 nM IgG-ADC	101.7	108.6	105.2	107.7	105.8	3.1
	10 nM Payload	12.3	15.2	12.5	13.0	13.3	1.3
WT	Control	99.8	95.7	103.9	100.5	100.0	3.4
	10 nM HER3-DXd	87.4	85.7	90.2	84.4	86.9	2.5
	10 nM Patritumab	103.3	107.4	112.5	113.0	109.0	4.6
	10 nM IgG-ADC	114.0	110.5	116.2	117.1	114.4	2.9
	10 nM Payload	15.0	14.5	15.0	14.4	14.7	0.3
V104L	Control	97.7	100.5	105.4	96.5	100.0	4.0
	10 nM HER3-DXd	90.1	90.7	91.9	94.1	91.7	1.8
	10 nM Patritumab	100.6	107.2	112.4	114.0	108.6	6.0
	10 nM IgG-ADC	105.4	112.5	120.0	112.4	112.6	6.0
	10 nM Payload	9.6	10.1	9.7	10.5	10.0	0.4
V104M	Control	99.1	97.8	105.9	97.2	100.0	4.0
	10 nM HER3-DXd	83.3	92.9	93.0	96.6	91.5	5.7

	10 nM Patritumab	94.6	103.7	106.3	107.7	103.1	5.9
	10 nM IgG-ADC	103.3	109.8	111.1	105.3	107.4	3.7
	10 nM Payload	13.8	15.4	15.1	13.5	14.5	0.9
A232V	Control	100.6	101.5	102.2	95.7	100.0	2.9
	10 nM HER3-DXd	83.9	85.4	90.2	84.4	86.0	2.9
	10 nM Patritumab	101.5	106.0	110.6	98.8	104.2	5.1
	10 nM IgG-ADC	107.8	106.5	114.5	109.3	109.5	3.5
	10 nM Payload	11.5	13.9	14.4	13.8	13.4	1.3
P262H	Control	102.6	99.3	96.3	101.8	100.0	2.8
	10 nM HER3-DXd	83.7	84.5	85.9	87.2	85.3	1.6
	10 nM Patritumab	99.0	104.7	103.1	110.5	104.3	4.8
	10 nM IgG-ADC	116.0	108.7	117.5	110.6	113.2	4.2
	10 nM Payload	14.3	15.7	15.0	15.6	15.1	0.6
G284R	Control	99.0	101.4	98.2	101.4	100.0	1.6
	10 nM HER3-DXd	81.4	87.3	85.0	85.6	84.8	2.5
	10 nM Patritumab	95.5	105.6	106.2	110.5	104.4	6.3
	10 nM IgG-ADC	105.5	115.3	115.2	116.1	113.0	5.1
	10 nM Payload	15.3	16.3	17.8	17.5	16.7	1.1
D297Y	Control	98.4	103.3	98.1	100.2	100.0	2.4
	10 nM HER3-DXd	83.5	87.4	83.7	87.9	85.6	2.3
	10 nM Patritumab	99.2	111.2	111.9	105.2	106.9	6.0
	10 nM IgG-ADC	109.7	104.2	110.1	117.9	110.5	5.6
	10 nM Payload	13.5	14.2	15.1	16.6	14.8	1.3

G325R	Control	100.8	102.4	99.4	97.4	100.0	2.1
	10 nM HER3-DXd	88.8	99.0	87.5	90.6	91.5	5.2
	10 nM Patritumab	100.5	106.4	109.7	109.8	106.6	4.4
	10 nM IgG-ADC	101.4	106.9	117.1	113.5	109.7	7.0
	10 nM Payload	17.2	18.6	17.7	18.0	17.9	0.6
T355I	Control	98.1	99.4	101.3	101.2	100.0	1.5
	10 nM HER3-DXd	87.5	94.0	93.8	93.5	92.2	3.2
	10 nM Patritumab	103.6	103.9	109.4	107.9	106.2	2.9
	10 nM IgG-ADC	107.3	113.2	116.3	117.2	113.5	4.5
	10 nM Payload	12.7	15.3	15.8	16.4	15.0	1.6
S846I	Control	96.1	98.9	100.9	104.0	100.0	3.3
	10 nM HER3-DXd	81.7	83.6	95.6	91.5	88.1	6.5
	10 nM Patritumab	102.1	109.4	108.3	102.4	105.5	3.8
	10 nM IgG-ADC	109.9	113.1	120.6	110.8	113.6	4.9
	10 nM Payload	14.1	15.8	15.7	12.9	14.6	1.4
E928G	Control	97.3	101.5	104.0	97.3	100.0	3.3
	10 nM HER3-DXd	81.9	84.6	90.7	87.8	86.2	3.8
	10 nM Patritumab	93.6	96.3	108.1	105.1	100.8	7.0
	10 nM IgG-ADC	102.6	104.1	110.3	112.2	107.3	4.7
	10 nM Payload	11.0	13.3	13.3	14.2	13.0	1.4

B) HER2 overexpression (+)

HER3	Test article	Cell viability (%)						
		#1	#2	#3	#4	#5	Mean	SD
EV	Control	98.3	93.8	105.6	103.2	99.1	100.0	4.6
	10 nM HER3-DXd	96.5	99.4	98.9	91.6	94.5	96.2	3.2
	10 nM Patritumab	98.4	92.9	105.1	110.4	105.3	102.4	6.8
	10 nM IgG-ADC	94.6	107.1	99.4	101.1	97.6	100.0	4.7
	10 nM Payload	15.4	19.7	12.0			15.7	3.9
WT	Control	99.6	103.4	102.7	93.6	100.8	100.0	3.9
	10 nM HER3-DXd	81.2	77.5	74.7	79.2	63.9	75.3	6.8
	10 nM Patritumab	93.2	96.7	95.1	96.7	98.6	96.1	2.0
	10 nM IgG-ADC	107.4	106.2	99.6	98.9	99.2	102.3	4.2
	10 nM Payload	13.6	13.0	12.8			13.1	0.4
V104L	Control	91.7	104.7	108.3	99.6	95.7	100.0	6.7
	10 nM HER3-DXd	75.8	80.2	83.1	79.4	72.6	78.2	4.1
	10 nM Patritumab	94.9	96.4	107.7	103.2	94.6	99.4	5.8
	10 nM IgG-ADC	100.4	99.0	103.9	92.0	94.6	98.0	4.7
	10 nM Payload	18.1	15.1	13.0			15.4	2.6
V104M	Control	99.2	98.2	98.2	100.6	103.8	100.0	2.3
	10 nM HER3-DXd	87.9	93.9	88.1	88.2	89.4	89.5	2.5
	10 nM Patritumab	99.2	101.0	101.8	103.1	99.5	100.9	1.6
	10 nM IgG-ADC	104.7	111.2	99.9	97.6	94.4	101.6	6.6
	10 nM Payload	20.5	20.2	20.6			20.4	0.2

A232V	Control	94.2	104.6	103.0	99.0	99.1	100.0	4.0
	10 nM HER3-DXd	77.9	87.2	83.1	79.5	69.4	79.4	6.6
	10 nM Patritumab	98.7	92.9	100.9	97.9	102.1	98.5	3.5
	10 nM IgG-ADC	100.0	107.4	102.3	99.8	95.2	100.9	4.4
	10 nM Payload	16.4	17.6	14.1			16.0	1.8
P262H	Control	101.6	100.2	96.9	102.8	98.6	100.0	2.4
	10 nM HER3-DXd	70.3	71.8	70.2	66.4	69.6	69.7	2.0
	10 nM Patritumab	97.7	90.8	104.5	98.1	100.6	98.4	5.0
	10 nM IgG-ADC	101.6	99.6	95.9	99.7	95.5	98.5	2.7
	10 nM Payload	14.4	14.8	14.1			14.4	0.3
G284R	Control	92.5	110.4	100.3	99.1	97.7	100.0	6.5
	10 nM HER3-DXd	68.4	77.3	73.1	73.8	71.1	72.8	3.3
	10 nM Patritumab	93.7	96.4	101.0	102.6	99.3	98.6	3.6
	10 nM IgG-ADC	108.8	107.3	105.4	103.1	94.0	103.7	5.8
	10 nM Payload	15.4	17.9	11.1			14.8	3.5
D297Y	Control	97.3	102.9	101.6	99.7	98.5	100.0	2.3
	10 nM HER3-DXd	64.3	66.7	59.6	66.3	53.8	62.2	5.4
	10 nM Patritumab	95.4	98.7	93.4	95.4	95.8	95.7	1.9
	10 nM IgG-ADC	103.2	103.6	100.2	98.2	96.6	100.4	3.1
	10 nM Payload	12.5	12.5	8.4			11.1	2.4
G325R	Control	99.9	97.0	98.0	100.3	104.8	100.0	3.0
	10 nM HER3-DXd	71.5	68.4	64.6	67.4	64.6	67.3	2.9
	10 nM Patritumab	100.7	97.1	106.1	101.6	96.5	100.4	3.9

	10 nM IgG-ADC	109.5	102.4	112.6	104.6	94.5	104.7	7.0
	10 nM Payload	12.8	14.5	14.6			14.0	1.0
T355I	Control	96.8	105.4	102.5	100.0	95.3	100.0	4.1
	10 nM HER3-DXd	69.9	74.2	72.7	72.3	68.8	71.6	2.2
	10 nM Patritumab	90.2	103.7	97.1	93.1	97.1	96.3	5.1
	10 nM IgG-ADC	96.3	101.0	96.5	99.4	95.7	97.8	2.3
	10 nM Payload	13.9	11.0	12.8			12.6	1.4
S846I	Control	99.1	96.5	97.9	101.3	105.2	100.0	3.4
	10 nM HER3-DXd	59.8	68.6	63.9	62.9	60.3	63.1	3.5
	10 nM Patritumab	97.7	92.9	91.1	96.0	101.5	95.8	4.1
	10 nM IgG-ADC	108.1	106.2	93.7	98.0	91.1	99.4	7.5
	10 nM Payload	14.6	14.0	14.3			14.3	0.3
E928G	Control	98.7	100.8	96.5	104.8	99.2	100.0	3.1
	10 nM HER3-DXd	68.5	65.2	68.4	70.5	61.8	66.9	3.4
	10 nM Patritumab	93.5	100.2	91.7	90.1	100.0	95.1	4.7
	10 nM IgG-ADC	100.4	106.9	93.7	105.7	92.1	99.8	6.7
	10 nM Payload	12.8	14.4	10.9			12.7	1.8
EV (HER2-)	Control	95.1	101.7	100.5	98.6	104.2	100.0	3.4
	10 nM HER3-DXd	108.2	104.2	100.6	101.4	106.3	104.1	3.2
	10 nM Patritumab	101.2	99.5	98.2	107.2	107.0	102.6	4.2
	10 nM IgG-ADC	107.4	108.6	104.7	97.4	102.2	104.0	4.5
	10 nM Payload	14.9	14.4	12.5			13.9	1.3
WT (HER2-)	Control	97.9	100.5	103.1	101.3	97.1	100.0	2.5

	10 nM HER3-DXd	87.1	83.6	80.9	71.2	76.2	79.8	6.2
	10 nM Patritumab	93.8	91.7	94.4	97.6	92.2	93.9	2.4
	10 nM IgG-ADC	110.0	100.9	99.3	102.2	95.6	101.6	5.3
	10 nM Payload	13.6	11.9	11.4			12.3	1.2

C) HER2 overexpression (+), Q809R

HER3	Test article	Cell viability (%)						
		#1	#2	#3	#4	#5	Mean	SD
EV	Control	99.1	97.8	101.0	99.8	102.3	100.0	1.7
	10 nM HER3-DXd	104.8	102.2	102.8	101.8	98.0	101.9	2.5
	10 nM Patritumab	99.1	104.9	104.9	110.8	104.6	104.9	4.1
	10 nM IgG-ADC	106.0	104.4	106.6	106.3	95.2	103.7	4.8
	10 nM Payload	24.9	22.2	19.3			22.2	2.8
WT	Control	101.2	98.0	99.9	101.2	99.7	100.0	1.3
	10 nM HER3-DXd	87.9	89.2	90.9	90.0	77.6	87.1	5.4
	10 nM Patritumab	97.8	107.8	109.9	110.6	107.6	106.8	5.1
	10 nM IgG-ADC	107.9	113.2	116.0	107.6	98.5	108.7	6.7
	10 nM Payload	20.0	17.6	15.9			17.8	2.0
Q809R	Control	100.0	100.6	101.3	99.3	98.8	100.0	1.0
	10 nM HER3-DXd	83.5	86.3	87.3	83.2	77.8	83.6	3.7
	10 nM Patritumab	98.0	101.7	99.2	102.3	101.0	100.4	1.8

	10 nM IgG-ADC	102.2	97.7	100.7	102.5	97.0	100.0	2.5
	10 nM Payload	20.2	20.2	18.7			19.7	0.9
EV (HER2-)	Control	98.4	100.1	101.1	100.5	99.8	100.0	1.0
	10 nM HER3-DXd	100.7	100.8	95.7	101.0	94.9	98.6	3.0
	10 nM Patritumab	98.9	100.7	100.8	104.1	103.8	101.7	2.2
	10 nM IgG-ADC	104.1	101.7	101.4	99.1	98.2	100.9	2.3
	10 nM Payload	20.3	19.2	17.3			18.9	1.5
WT (HER2-)	Control	95.1	99.9	100.7	102.8	101.4	100.0	2.9
	10 nM HER3-DXd	91.9	89.8	88.7	86.5	83.5	88.1	3.2
	10 nM Patritumab	100.2	99.3	102.8	103.3	103.4	101.8	1.9
	10 nM IgG-ADC	103.7	104.9	103.1	100.5	97.9	102.0	2.8
	10 nM Payload	19.8	19.8	17.6			19.1	1.3
